# Multiple Acid Sensors Control *Helicobacter pylori* Colonization of the Stomach

**DOI:** 10.1371/journal.ppat.1006118

**Published:** 2017-01-19

**Authors:** Julie Y. Huang, Emily Goers Sweeney, Karen Guillemin, Manuel R. Amieva

**Affiliations:** 1 Department of Microbiology and Immunology, Stanford University School of Medicine, Stanford, California, United States of America; 2 Institute of Molecular Biology, University of Oregon, Eugene, Oregon, United States of America; 3 Humans and the Microbiome Program, Canadian Institute for Advanced Research, Toronto, Ontario, Canada; 4 Department of Pediatrics, Stanford University School of Medicine, Stanford, California, United States of America; Osaka University, JAPAN

## Abstract

*Helicobacter pylori’s* ability to respond to environmental cues in the stomach is integral to its survival. By directly visualizing *H*. *pylori* swimming behavior when encountering a microscopic gradient consisting of the repellent acid and attractant urea, we found that *H*. *pylori* is able to simultaneously detect both signals, and its response depends on the magnitudes of the individual signals. By testing for the bacteria’s response to a pure acid gradient, we discovered that the chemoreceptors TlpA and TlpD are each independent acid sensors. They enable *H*. *pylori* to respond to and escape from increases in hydrogen ion concentration near 100 nanomolar. TlpD also mediates attraction to basic pH, a response dampened by another chemoreceptor TlpB. *H*. *pylori* mutants lacking both TlpA and TlpD (Δ*tlpAD*) are unable to sense acid and are defective in establishing colonization in the murine stomach. However, blocking acid production in the stomach with omeprazole rescues Δ*tlpAD’s* colonization defect. We used 3D confocal microscopy to determine how acid blockade affects the distribution of *H*. *pylori* in the stomach. We found that stomach acid controls not only the overall bacterial density, but also the microscopic distribution of bacteria that colonize the epithelium deep in the gastric glands. In omeprazole treated animals, bacterial abundance is increased in the antral glands, and gland colonization range is extended to the corpus. Our findings indicate that *H*. *pylori* has evolved at least two independent receptors capable of detecting acid gradients, allowing not only survival in the stomach, but also controlling the interaction of the bacteria with the epithelium.

## Author Summary

*Helicobacter pylori* is a bacterium that chronically infects the stomachs of 50% of humans, and infection can lead to serious diseases like peptic ulcers and stomach cancer. To survive, *H*. *pylori* rapidly senses acid and swims away to the protective mucus layer covering the stomach surface. The bacteria also burrow deep into the glands of the stomach through their narrow fissures and channels, and live in close contact with the cells lining the stomach. We report here that two *H*. *pylori* chemoreceptors, TlpA and TlpD, are the dominant acid sensors enabling *H*. *pylori* to discern and respond to minute changes in acid levels. *H*. *pylori* mutants lacking both TlpA and TlpD are unable to sense acid and are severely impaired in their survival in the murine stomach. By treating animals with omeprazole, a drug that blocks acid production, we restored the ability of the acid-sensor mutant to survive in the stomach. In addition, we found that blocking stomach acid production extended the range, distribution, and density of *H*. *pylori* living deep in the gastric glands. Our study provides new insights into *H*. *pylori’s* acid sensing machinery and how manipulation of acid gradients controls *H*. *pylori’s* localization and survival in the stomach.

## Introduction

*Helicobacter pylori* is a bacterium that has co-evolved with humans since the origin of the human species [1, 2]. This intimate association with the human host has allowed it to develop a number of survival strategies to persist in one of the most challenging environments in the human body—the stomach. *H*. *pylori’s* survival relies on its ability to avoid the microbicidal effects of stomach acid. *H*. *pylori* can withstand acidic conditions for short periods of time due to its urease enzyme which degrades urea into ammonium and bicarbonate to buffer the cytoplasm and periplasm [3–6]. Another important strategy is to utilize chemotaxis to locate and swim to the gastric epithelium where the pH is near neutral due to the overlying protective mucus layer.

The bacteria colonize a narrow niche within 25 microns of the surface of the gastric epithelium where they are either found actively swimming in the mucus or directly adhered to epithelial cells [7, 8]. The attached bacteria utilize virulence factors to obtain essential nutrients from the host and replicate on the cell surface to form cell-associated microcolonies [9, 10]. We recently reported that a subpopulation of cell-associated *H*. *pylori* is found deep in the antral glands in direct contact with gastric progenitor cells and stem cells [11]. The gland-associated *H*. *pylori* induce the expansion and proliferation of stem cells, alter stem cell gene expression, and lead to gland hyperplasia [11]. We hypothesize that the factors that control *H*. *pylori’s* ability to colonize the gastric glands will help explain *H*. *pylori’s* ability to persist long-term in the stomach and to cause gastric diseases.

Despite living in the stomach, *H*. *pylori* is not an acidophile and swims away from hydrochloric acid (HCl). The acid secreted into the stomach lumen by parietal cells in the corpus forms gradients that keep the bacteria close to the gastric epithelium [8]. Previous studies have reported that the chemoreceptor responsible for sensing HCl as a repellent is TlpB [12, 13]. This chemoreceptor has been shown to detect auto-inducer 2 as a repellent as well [14]. We recently reported that TlpB can also sense chemoattractants, since it is a high affinity chemoreceptor for urea that allows *H*. *pylori* to sensitively detect and swim towards urea emanating from the gastric epithelium [15].

In this study, we initially proceeded to investigate how *H*. *pylori* may be sensing both a repellent and an attractant through TlpB. Using a previously developed videomicroscopy method that visualizes and films bacterial chemotactic responses to chemical gradients in real time [7, 15], we discovered that *H*. *pylori* mutants lacking TlpB (Δ*tlpB*) are not defective in detecting and swimming away from HCl gradients. Instead, we identified TlpA and TlpD as independent acid sensors with different sensitivities to HCl. We also found that TlpD allows *H*. *pylori* to chemotax towards less acidic and even basic pH environments, and this response is dampened by TlpB.

Using a murine model of infection in the stomach, we discovered that the double mutant lacking TlpA and TlpD (Δ*tlpAD*) is about 100-fold defective in its ability to colonize the stomach compared to wild-type *H*. *pylori*. However, treatment with the proton-pump inhibitor omeprazole raises the gastric pH and partially rescues the Δ*tlpAD* mutant’s defect, allowing it to reach significantly higher bacterial numbers in the stomach. We also observed that omeprazole treatment promotes wild-type *H*. *pylori’s* colonization of the gastric glands and extends its range of glandular colonization from the antrum into the glands of the corpus. Despite the higher loads of Δ*tlpAD H*. *pylori* in omeprazole-treated animals, the mutant is unable to colonize the gastric glands to the same levels as wild-type, suggesting that these two chemoreceptors are important in establishing colonization deep in the gastric glands.

Our study has identified two new roles for *H*. *pylori’s* chemoreceptors as acid sensors and demonstrate that *H*. *pylori*’s ability to detect and respond to the acid gradient is important for its localization within the stomach, its interaction with the glandular epithelium, and its survival *in vivo*.

## Results

### *H*. *pylori* mutants lacking TlpB swim away from acid gradients

*H*. *pylori* encounters many chemical gradients in the stomach that serve as cues to identify microniches that are safe for colonization. It must be able to integrate both repellents and attractants to optimize its chemotactic response. Since *H*. *pylori*’s TlpB chemoreceptor has been reported to detect HCl as a repellent [12, 13], and we found that it detects urea as a high-sensitivity attractant [15], we wondered how *H*. *pylori* would respond if it were simultaneously exposed to both urea and HCl gradients emanating from one point source.

**Fig 1:**
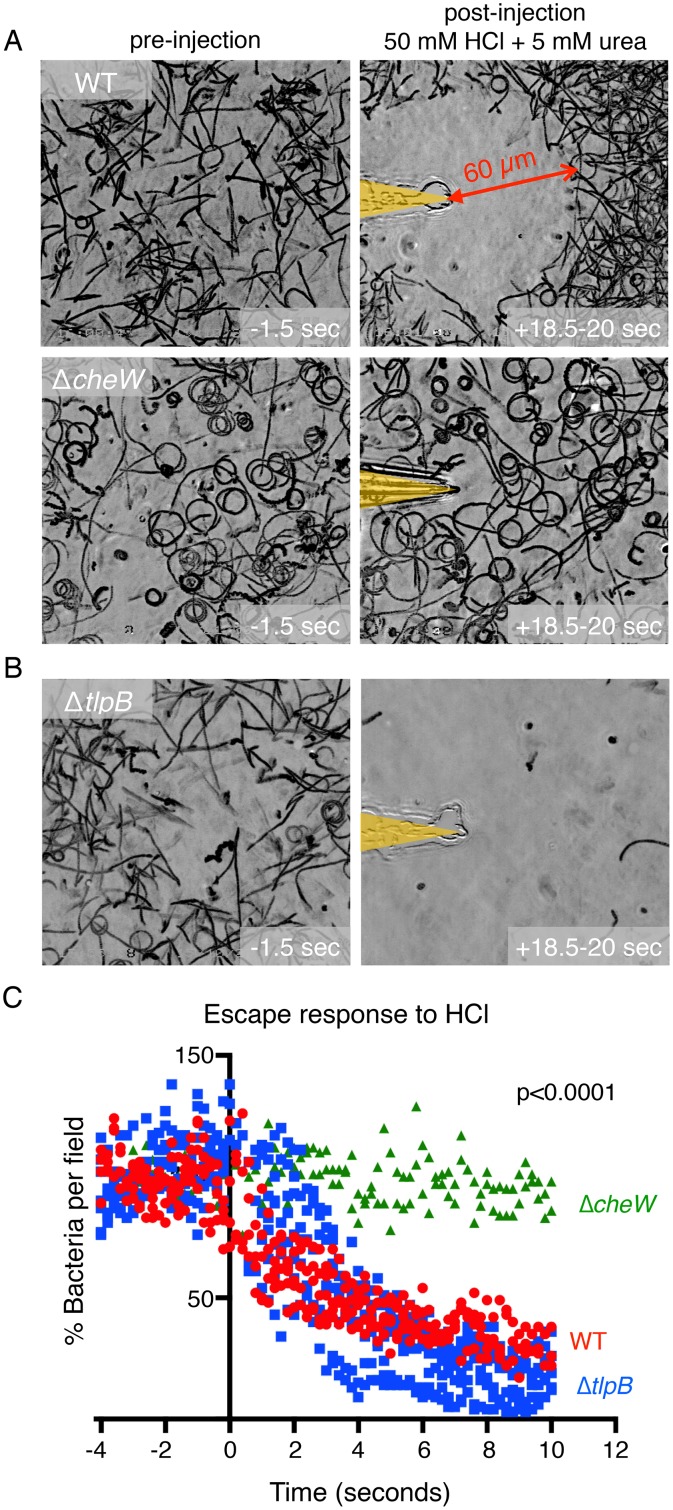
*H*. *pylori* simultaneously responds to chemoattractants and repellents and does not require TlpB to swim away from an acid gradient. (**A**) Still images of bacterial motility traces (lasting 1.5 seconds) of PMSS1 wild-type (WT) *H*. *pylori* and Δ*cheW H*. *pylori* within the field of view 1.5 seconds before and 18.5 seconds after exposure to a combined gradient of 5 mM urea and 50 mM hydrochloric acid. The position of the microinjection needle tip is highlighted in yellow. (**B**) Still images of bacterial motility traces (lasting 1.5 seconds) of Δ*tlpB H*. *pylori* within the field of view 1.5 seconds before and 18.5 seconds after exposure to a gradient of 5 mM urea and 50 mM hydrochloric acid. (**C**) Quantification of the responses of WT *H*. *pylori* vs. Δ*tlpB* vs Δ*cheW* to a 100 mM hydrochloric acid gradient. Each point represents the percent of swimming bacteria remaining in the field of view at each time point in the digitized video microscopy movie frames. Points for three to five movies are plotted per condition and grouped by color as indicated. Time zero is defined as the moment the needle is introduced and the gradient is initiated. p-value for scatter plot indicates significance of time by group interaction via a 2-way repeated measures ANOVA.

We used our previously described microgradient chemotaxis assay [7, 15] to record the swimming responses of a live culture of *H*. *pylori* (strain PMSS1) exposed to a microscopic gradient of a mixture of 50 mM HCl and 5 mM urea emanating from the tip of a microinjection needle. Prior to the assay, *H*. *pylori* were grown in Brucella broth with 10% FBS (BB10), pH 6.7–6.8 to an OD_600_ of 0.3. During culture, bacterial urease depletes urea from the surrounding medium (S1A Fig) and the pH remains at a range of 6.6–6.74 (S1B Fig). A 270 μl volume of media with motile bacteria is placed onto a coverslip chamber and a microinjection needle is then rapidly inserted via a micromanipulator into the viewing field to produce a microscopic gradient of chemoeffectors. Using this method, we observed that *H*. *pylori* are attracted to the urea in the gradient until they reach a boundary approximately 60 micrometers away from the needle tip (Fig 1A). Within this boundary is a zone of clearance avoided by the bacteria, representing a threshold concentration of acid that acts as a chemorepellant. When bacteria swim into this zone of clearance, they quickly stop, reverse direction, and swim away (S1 Movie). As a negative control, we tested a chemotaxis null mutant Δ*cheW H*. *pylori* for its response to the same mixed microgradient. The density of swimming Δ*cheW H*. *pylori* remained constant throughout the field (Fig 1A) consistent with this mutant’s inability to respond to either the attractant or the repellent. These observations indicate that *H*. *pylori* is capable of simultaneously sensing and integrating multiple signals from one point source. To test whether the chemotactic response is dependent on the magnitude of these chemical gradients, we altered the concentration of acid within the needle while maintaining the same concentration of urea. We found that, indeed, the bacteria respond by increasing the distance of the boundary between repulsion and attraction and the needle tip as the concentration of HCl increases (S2 Fig).

Because *H*. *pylori* abundantly expresses a urease enzyme that degrades urea into ammonia to buffer its cytoplasmic and periplasmic pH, we wondered whether urease may play a role in acid sensing. We first tested a urease mutant (Δ*ureAB*) for its response to a solution of urea and HCl. Unlike the WT *H*. *pylori* culture, which is depleted of urea due to urease activity, the Δ*ureAB* culture contains levels of urea in the medium comparable to fresh media (S1A). We observed that Δ*ureAB* cleared the field of view completely (S3A Fig), indicating that it was unable to sense urea because the surrounding urea in the medium interferes with chemotactic sensing as we had previously reported [15] but was able to sense acid. We further tested for Δ*ureAB’s* response to a solution of 100 mM HCl in water (no urea). We found that it cleared the field of view like wild-type (S3B Fig). These results suggest that urease activity is not required for acid sensing.

Next, we tested Δ*tlpB H*. *pylori*’s response to this mixed solution. As the urea and acid sensor, we predicted that this mutant would respond to neither compound and that its swimming behavior would be like that of Δ*cheW H*. *pylori*. We were surprised to find instead that Δ*tlpB H*. *pylori* rapidly swims away and clears from the field of view like Δ*ureAB* (Fig 1B, S3A Fig, and S1 Movie). This observation that the bacteria swim away from the urea and acid microgradient suggests that the Δ*tlpB* mutant is unable to detect urea as we had previously reported [15] but is able to detect HCl as a repellent.

We proceeded to test Δ*tlpB’s* response to a solution of HCl in water without urea. We found that, indeed, Δ*tlpB* efficiently swims away from an acid gradient (Fig 1C and S2 Movie). We plotted the bacterial density in the viewing field every fifth of a second before and after introduction of the needle tip injecting HCl, and by ten seconds post-exposure to the acid gradient, the Δ*tlpB* mutant has mostly cleared from the field of view (Fig 1C). Interestingly, we note from the clearance curves (Fig 1C) that Δ*tlpB* clears from the field of view faster than wild-type upon acid exposure. This result suggests that TlpB does affect *H*. *pylori’s* response to acid since Δ*tlpB* has response kinetics different from wild-type. To verify that the Δ*tlpB* response to acid is not specific to a particular *H*. *pylori* strain, we constructed Δ*tlpB* mutants in other strains of *H*. *pylori* and tested them in the same assay. We found that the Δ*tlpB* mutants in all strains tested (strains G27, SS1, PMSS1 (a second independent clone), and 7.13) are still able to respond to an acid gradient like their wild-type counterparts by swimming away upon exposure to HCl and maintain the fast clearing phenotype (S4 Fig). This result indicates that Δ*tlpB’s* response to HCl is not strain specific. For the rest of the experiments we use *H*. *pylori* PMSS1 as the strain background.

### *H*. *pylori* expresses two chemoreceptors that are each acid sensors

**Fig 2:**
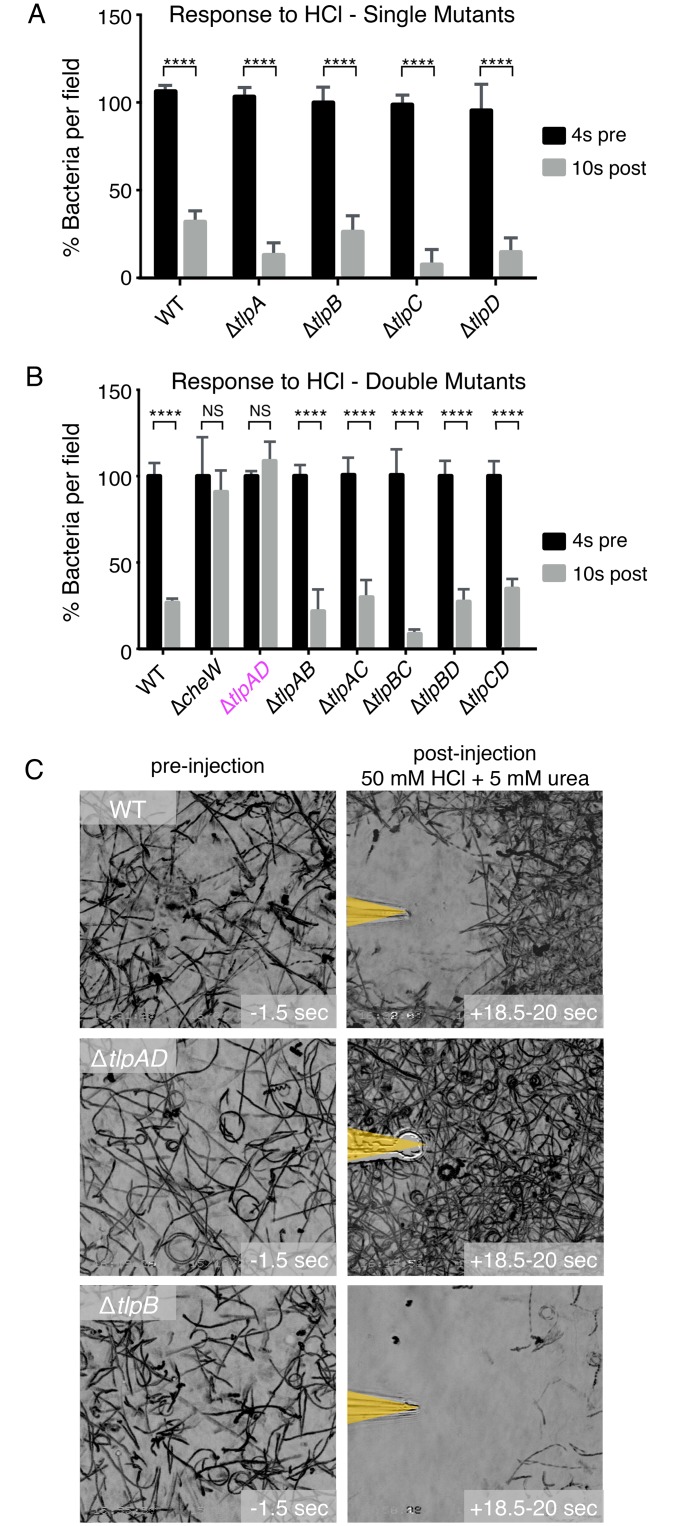
*H*. *pylori* expresses two chemoreceptors that rapidly sense acid gradients. (**A**) Quantification of the responses of wild-type (WT) *H*. *pylori* vs. Δ*tlpA*, Δ*tlpB*, Δ*tlpC*, Δ*tlpD* to a 100 mM hydrochloric acid gradient. Temporal data of motility responses were collected by videomicroscopy and quantified as in Fig 1C. The difference in bacterial density within the field of view at two time points, four seconds before the acid gradient (pre) and ten seconds after exposure to the acid gradient (post) is represented by bar graphs. (**B**) Quantification of the responses of WT *H*. *pylori* vs. Δ*cheW*, Δ*tlpAD*, Δ*tlpAB*, Δ*tlpAC*, Δ*tlpBC*, Δ*tlpBD*, Δ*tlpCD* to a 100 mM hydrochloric acid gradient. Each bar represents the percent of bacteria in the field of view at four seconds pre-exposure and 10 seconds post-exposure to the gradient. (**C**) Still images of bacterial motility traces (lasting 1.5 seconds) of WT, Δ*tlpAD*, and Δ*tlpB H*. *pylori* within the field of view before and after exposure to a gradient of 5 mM urea and 50 mM hydrochloric acid. (n = 3 movies per condition). Bars represent the mean. Error bars represent s.d. NS indicates no statistical significance, **** *P* < 0.0001 (2-way repeated measures ANOVA).

Our finding that Δ*tlpB* responds to an HCl gradient suggests that other chemoreceptors may be acid sensors. We tested the response of mutants lacking each of the other three chemoreceptors, Δ*tlpA*, Δ*tlpC*, Δ*tlpD*, by videomicroscopy in the same assay. To simplify the comparisons of the movies showing the escape from acid of different mutants, we graphed the percent of bacteria present in the viewing field 4 seconds before and 10 seconds after exposure to the acid gradient (Fig 2A). We found that each mutant was still able to respond and swim away from acid, indicating that either there is a novel unidentified chemoreceptor that senses acid or there are multiple chemoreceptors that can function redundantly in sensing acid.

To test the hypothesis that there may be redundancy in acid-sensing chemoreceptors, we made mutants lacking two of the four chemoreceptors in all possible combinations. We discovered that of all the six combinations of chemoreceptor knock-outs, only the mutant lacking both TlpA and TlpD (Δ*tlpAD H*. *pylori*) lost the ability to respond to HCl gradients (Fig 2B and S3 Movie). This result indicates that TlpA and TlpD function as acid sensors, each capable of compensating for the loss of the other in acid sensing.

Furthermore, when we tested for the response of Δ*tlpAD H*. *pylori* to the solution containing a mixture of urea and HCl, we observed that the bacteria are attracted towards the needle tip with no zone of clearance (Fig 2C). This result indicates that the Δ*tlpAD* mutant can detect and respond to urea as an attractant (through TlpB), but is unable to detect the HCl as a repellent. This result suggests that TlpB is not sufficient to sense acid in our assay, and TlpA and TlpD are acid sensors that detect acid gradients.

We next asked whether the response to acid is a response to low pH or a response specifically to HCl. To determine this, we tested the responses of WT, Δ*tlpA*, Δ*tlpB*, Δ*tlpD*, and Δ*tlpAD* to sulfuric acid (H_2_SO_4_) and phosphoric acid (H_3_PO_4_). As with HCl, we observed that *H*. *pylori* responds to both of these acids as repellents and requires either TlpA or TlpD for the response (S5 Fig). This result suggests that these two chemoreceptors allow *H*. *pylori* to sense and escape from conditions of low pH.

### TlpD is more sensitive than TlpA in detecting hydrochloric acid

Several chemoeffectors have been described for TlpA and TlpD. The TlpA receptor was reported to mediate attraction to arginine and bicarbonate [16]. The TlpD chemoreceptor was reported to mediate repulsion from conditions that induce low-energy in the bacterium [17] or conditions that promote oxidative stress [18, 19]. These conditions may be triggered by low pH. One key difference between TlpA and TlpD is the location of these chemoreceptors within the bacterium. The TlpD chemoreceptor is cytoplasmic as it lacks a transmembrane domain [17] whereas the sensing domain of TlpA is periplasmic like that of the TlpB and TlpC chemoreceptors. Therefore, TlpD may be sensing changes in external pH indirectly. Given the difference in the location of these two chemoreceptors, we wondered if the two receptors may have distinguishable responses to the same HCl gradient.

**Fig 3:**
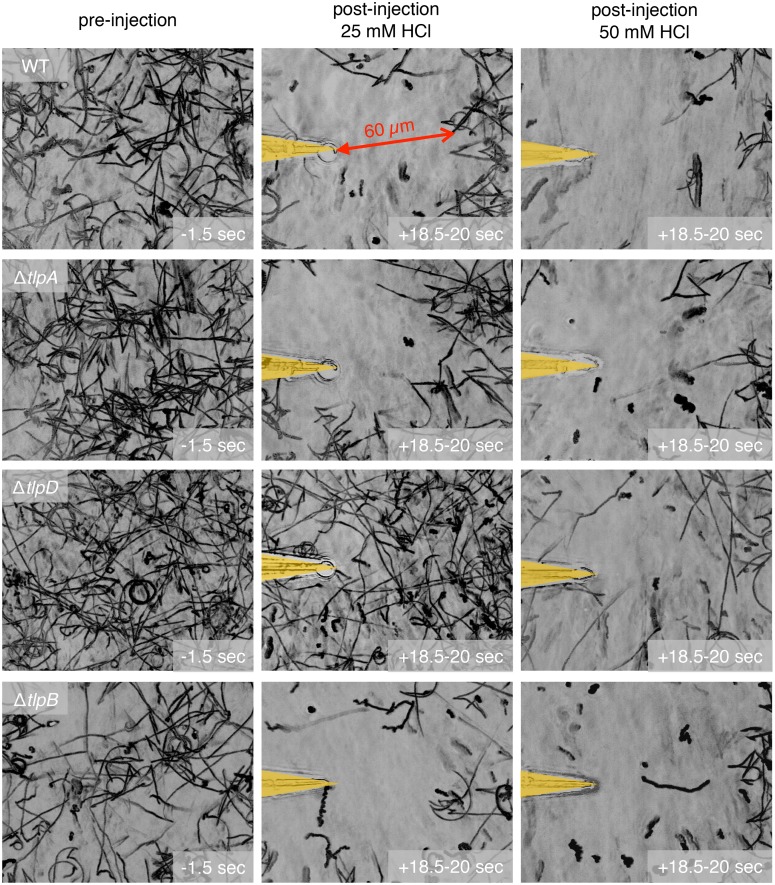
TlpD is more sensitive to a hydrochloric acid gradient than TlpA. Still images of bacterial motility traces (lasting 1.5 seconds) of wild-type *H*. *pylori* vs. Δ*tlpB* vs. Δ*tlpA* vs. Δ*tlpD* before (panels in left column) and after exposure to a gradient of either 25 mM (panels in middle column) or 50 mM hydrochloric acid (panels in right column). The positions of the needle tips are marked in yellow.

To assess if there is a difference in sensitivity for detecting HCl between the two chemoreceptors, we determined the threshold concentration of HCl necessary to elicit a response (arbitrarily defined as a 60 microns clearance zone from the point source) for each mutant. We empirically determined that 25 mM HCl loaded in the microinjection needle is the minimum concentration required to repel wild-type *H*. *pylori* from the point source by 60 microns (Fig 3). Δ*tlpA H*. *pylori*, like wild-type, responds to a minimal effective concentration of 25 mM HCl while Δ*tlpD H*. *pylori* requires 50 mM HCl to respond (Fig 3). It is worth noting, however, that the actual minimal effective concentration of HCl that *H*. *pylori* is capable of detecting is markedly below 25 mM, since only a minute volume of HCl on the order of 1 picoliter/minute is injected into the solution, and the culture medium surrounding the bacteria consists of Brucella broth with 10% fetal bovine serum, which has a buffering capacity that we empirically determined to be about 4,000 fold greater than water (S6 Fig). Thus the gradient of free hydrogen ions would drop rapidly away from the needle tip. Since we observe the bacteria responding 60 microns away from the point source, the change in HCl concentration that the bacteria can sense is substantially less than the concentration of the solution in the needle. Indeed, when we expose the bacteria to a gradient starting from 15 mM HCl at the needle tip, we also see the bacteria being repelled but at approximately 30 microns away from the point source (S7 Fig).

Since *H*. *pylori* is exposed to both urea and HCl in vivo, we wondered whether urea sensing would affect the sensitivities of TlpA and TlpD in sensing acid. We exposed Δ*tlpA* and Δ*tlpD* to a gradient of a mixture of 5 mM urea and 50 mM HCl in the microinjection needle. We observed that both mutants are attracted to the urea but form a zone of clearance as they sense HCl like wild-type *H*. *pylori*. However, with urea present we could elucidate small differences in sensitivity because the attraction to urea highlights TlpD’s ability to detect lower concentrations of acid than TlpA. This is illustrated by the Δ*tlpA* mutant remaining at a farther distance away from the acid point source at the needle tip compared to Δ*tlpD* (S8 Fig). This result indicates that urea sensing does not alter the sensitivity hierarchy of TlpD and TlpA in acid sensing.

### *H*. *pylori* senses and responds to both acidic and basic changes in pH with nanomolar sensitivity

As chemotaxis is a result of detecting a change in the concentration of a chemical, we next sought to determine the smallest change in hydrogen ion concentration ([H+]) that *H*. *pylori* is able to detect and respond to. By comparing the pH of the Brucella broth culture medium in which the bacteria are grown with the pH of a buffer solution that elicits a repulsion response, we determined the smallest difference in [H+] that *H*. *pylori* is capable of detecting. To more accurately control the range of [H+] concentrations experienced by the bacteria, we loaded the microinjection needle with 1M phosphate buffer solutions with defined pHs made by combining different proportions of dibasic and monobasic phosphate solutions. We measured the pH of the Brucella broth medium that the bacteria are grown in to be pH 6.7 (S1B Fig). We then tested wild-type bacteria’s response to buffer solutions ranging from pH 4.2 to 7.1.

**Fig 4:**
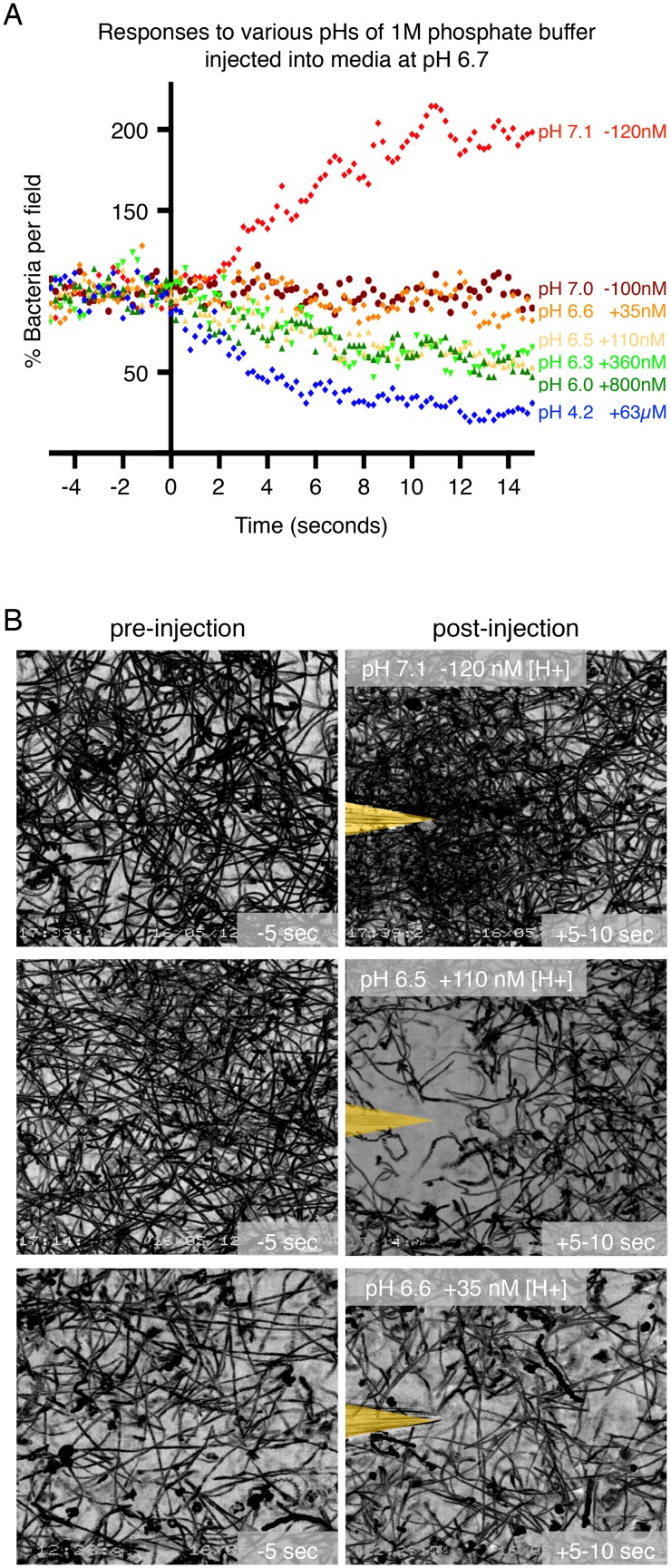
*H*. *pylori* can respond to acidic and basic changes in pH with nanomolar sensitivity. (**A**) Quantification of the responses of wild-type (WT) *H*. *pylori* to phosphate buffer solutions with pH ranging from 4.2 to 7.1. The addition (+) or depletion (-) of hydrogen ions relative to the hydrogen ion concentration in the culture medium (pH 6.7) is indicated next to the pH of the buffer solution introduced. Each point represents the percent of swimming bacteria remaining in the field of view at each time point in the digitized video microscopy movie frames. Points for one movie are plotted per condition. Time zero is defined as the moment the needle is introduced and the gradient is initiated. (**B**) Still images of bacterial motility traces (lasting 5 seconds) of WT *H*. *pylori* before (panels in left column) and after injection of a phosphate buffer solution with pH 7.1, 6.5 or 6.6 (panels in right column). The pH of the culture medium was 6.7. The positions of the needle tips are marked in yellow.

As shown in Fig 4A, buffers with pH 4.2, 6.0, 6.3, and 6.5 all elicited an escape response in the microgradient assay. The increase in [H+] between the solution released from the microinjection needle and the culture medium was approximately 63 μM, 800 nM, 360 nM, and 110 nM, respectively. For the lower pH measurements between pH 4 and pH 6 we also confirmed our results using citrate buffer, which buffers acidic pH more effectively than phosphate buffer (S9 Fig). Phosphate buffer solutions of pH 6.6 and 7.0, which locally changed the [H+] by + 35 nM or -100 nM, did not elicit a response, indicating that the differences were below the limit of detection of the receptors. Unexpectedly, a buffer solution with pH 7.1, (about -120nM change in [H+]) elicited an opposite response with bacteria attracted and forming a swarm at the needle tip (Fig 4B and S4 Movie). Thus, we discovered that *H*. *pylori* can sense and respond to small changes in both acidic and basic pH when the change in [H+] is greater than 100 nM.

### TlpA and TlpD both sense acidic pH while TlpD also detects basic pH

**Fig 5:**
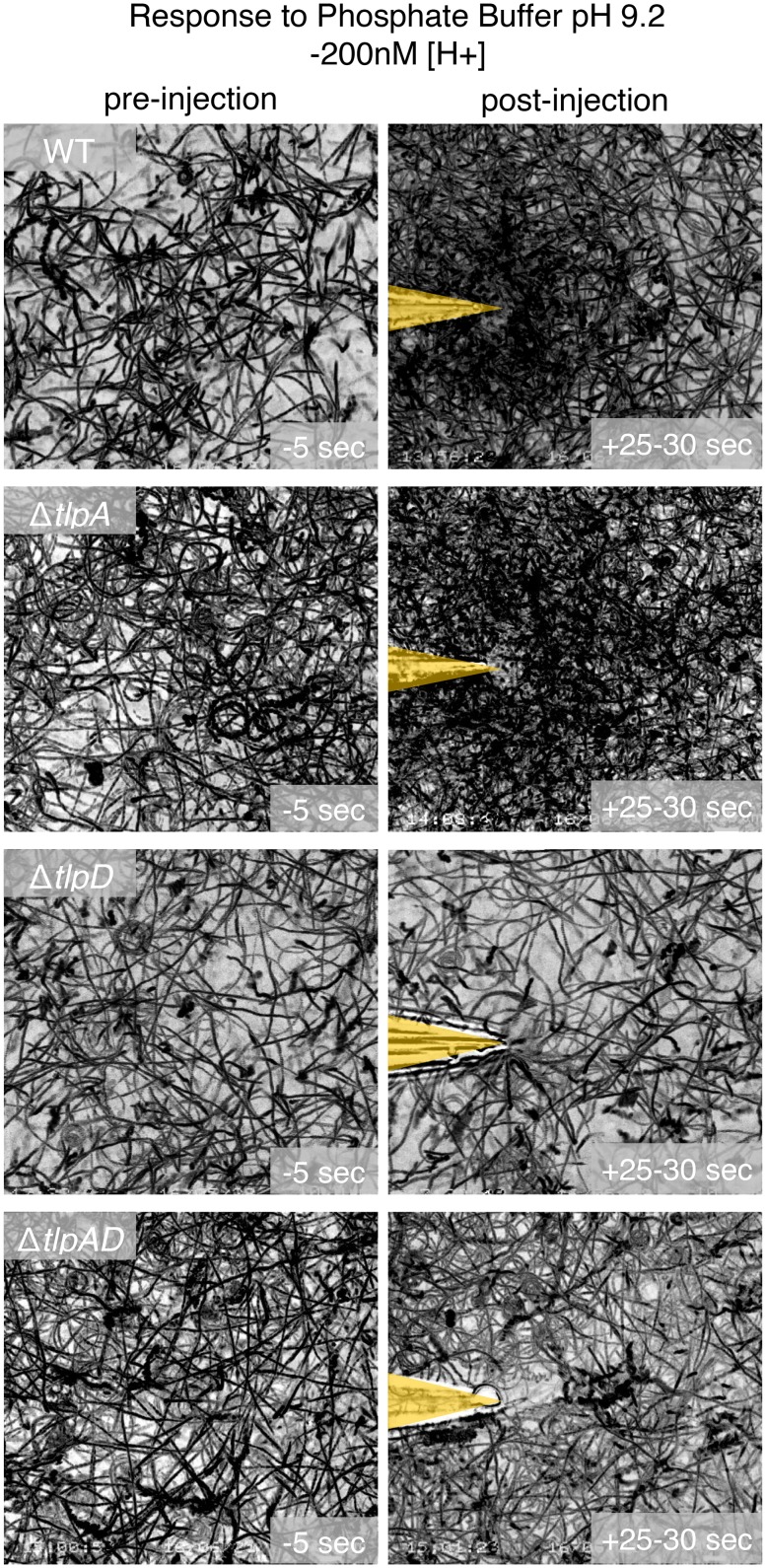
*H*. *pylori* attraction to basic changes in pH is mediated through TlpD. Still images of bacterial motility traces (lasting 5 seconds) of wild-type *H*. *pylori* vs. Δ*tlpA* vs. Δ*tlpD* vs. Δ*tlpAD* before (panels in left column) and after injection of a phosphate buffer solution with pH 9.2 (panels in right column). The pH of the culture medium was 6.7. The positions of the needle tips are marked in yellow.

To further characterize wild-type *H*. *pylori*’s response to more basic environments, we also tested phosphate buffers with pH 7.25 and 9.2. We found that *H*. *pylori* is attracted to basic pH solutions (S10 Fig). By testing the single chemoreceptor mutants, we identified TlpD as the necessary receptor for attraction to higher pH (Fig 5 and S5 Movie). The other receptors, including TlpA, were not necessary. To confirm that the TlpD-dependent attraction is a response to high pH rather than to a specific basic solution, we tested for *H*. *pylori’s* response to 40 mM sodium hydroxide (pH 12.6). We also observed *H*. *pylori* being attracted to sodium hydroxide, and the response was dependent on TlpD (S11 Fig). Taken together these results suggest that TlpD mediates both repulsion from lower pH and attraction to higher pH while TlpA only detects and mediates repulsion to lower pH.

### TlpB alters the response kinetics and sensitivity to changes in pH

We wondered whether the TlpB-dependent defect in acid sensing previously reported and our observation of faster clearance rates of Δ*tlpB* mutants compared to wild-type may be due to an effect of TlpB in modulating the sensitivity to pH of the other chemoreceptors. In order to see if lacking TlpB enhances the sensitivity to acid, we tested for the response of Δ*tlpB H*. *pylori* to low concentrations of HCl and found that the minimal effective concentration of HCl in the needle required to elicit an escape response is the same as that of wild-type at 25 mM HCl (Fig 3). We also tested the responses of the TlpA and TlpD chemoreceptors in the absence of TlpB by determining the sensitivities of the Δ*tlpAB* and Δ*tlpBD* double mutants to acid gradients. We found them to have the same sensitivity to HCl as Δ*tlpA* and Δ*tlpD*, respectively (S7 Fig).

However, when we tested for *H*. *pylori’s* response to higher pH, we noted that Δ*tlpB’s* attraction was always more pronounced than wild-type with each higher pH solution (S10 Fig); the attraction of Δ*tlpB* was faster, and the concentration of bacteria around the needle tip was denser than that of wild-type. This observation led us to hypothesize that TlpB may be modulating *H*. *pylori's* response to basic pH. We tested for the mutant’s response to a gradient formed by phosphate buffer at pH 7.0 where wild-type *H*. *pylori* does not show an attraction. The Δ*tlpB* mutant is still able to sense and respond to the solution at pH 7.0 (-100nM change in [H+]) (S10 Fig). This result suggests that lacking TlpB increases the bacteria's sensitivity and attraction to higher pH, and thus TlpB may be modulating the pH responses mediated through TlpD.

The mechanism by which TlpB influences pH sensing is unknown. We tested whether TlpB alters the expression levels of TlpA or TlpD, thereby resulting in a change in acid response kinetics. We performed a Western blot to assess expression levels of the chemoreceptors in the single knock-out mutants and Δ*tlpAD H*. *pylori*. We found that the expression levels of the remaining chemoreceptors were not affected by the loss of the TlpA, TlpB, or TlpD receptors (S12 Fig).

Our data show that TlpB is not sufficient for sensing acidic pH, but it does alter the kinetics of *H*. *pylori’s* response to changes in pH. Taken together, our data suggest that the mechanism of pH sensing in *H*. *pylori* is complex, involving multiple chemoreceptors. The difference in sensitivity between TlpA and TlpD and the role of TlpB in modulating pH responses allows *H*. *pylori* to discern even small changes in local pH gradients in the nanomolar range. This may have critical implications for *H*. *pylori’s* survival in the stomach.

### Loss of both TlpA and TlpD creates a deficiency in *H*. *pylori’s* ability to colonize the stomach, and this deficiency can be rescued by inhibiting acid production in the stomach

**Fig 6:**
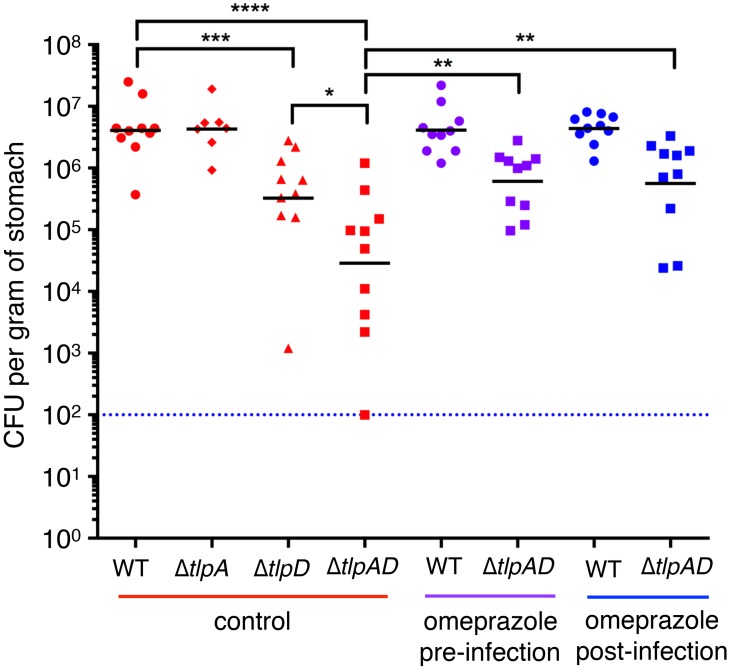
TlpA and TlpD are important in establishing colonization in acidic conditions in a murine model of gastric infection. Acid suppression rescues Δ*tlpAD* colonization defect. Recovered colony forming units (CFU) of *H*. *pylori* per gram of stomach tissue after a 2-week infection with wild-type (WT) *H*. *pylori*, Δ*tlpA H*. *pylori*, Δ*tlpD H*. *pylori*, or Δ*tlpAD H*. *pylori* (n = 10 mice for WT *H*. *pylori*, Δ*tlpAD H*. *pylori*, and Δ*tlpD H*. *pylori*; n = 7 for Δ*tlpA H*. *pylori*). For the experimental groups, omeprazole was administered in the drinking water 3 days prior to infection and throughout the course of the infection (purple), or for 1 week beginning 1 week after infection with *H*. *pylori* (blue). Blue dotted line indicates the limit of detection. Bars represent the geometric mean. * *P* < 0.05, ** *P* < 0.01, *** *P* < 0.001, **** *P* < 0.0001 (Mann Whitney test).

Given the importance of avoiding the acidic lumen of the stomach environment, we next investigated whether Δ*tlpAD H*. *pylori* would be able to colonize the stomachs of mice. We infected C57Bl/6 mice with either wild-type *H*. *pylori* strain PMSS1 or the isogenic mutants Δ*tlpA*, Δ*tlpD*, or Δ*tlpAD*. After two weeks of infection, a time point in which wild-type *H*. *pylori* has established stable colonization [7, 20], we harvested the stomachs and assessed colonization densities by colony-forming units (CFU) per gram of stomach tissue. We found that the Δ*tlpA* mutant was not defective in colonization with similar CFU counts as wild-type bacteria. However, the Δ*tlpD* mutant had a 10-fold defect as had been previously reported [20], and the Δ*tlpAD H*. *pylori* mutant was about 100-fold defective in establishing colonization (Fig 6). We hypothesized that without the ability to respond to acid gradients in the stomach, the Δ*tlpAD H*. *pylori* is deficient in avoiding the microbicidal HCl. We wondered if pharmacologic inhibition of acid secretion in the stomach would improve Δ*tlpAD H*. *pylori’s* survival.

We maintained two experimental groups to test this hypothesis. One group of animals was treated with the proton-pump inhibitor omeprazole for three days prior to infection to raise the gastric pH before infection (S13 Fig), and the animals continued to receive omeprazole throughout the course of the 2-week infection. This experimental group allowed us to observe how the gastric pH experienced by the bacteria upon entering the stomach affects the bacteria’s survival in the stomach. The second group of animals was infected with *H*. *pylori* for a week before treatment with omeprazole throughout the final week of infection. This group represents the more common clinical scenario in which humans with an established *H*. *pylori* infection may take proton-pump inhibitors like omeprazole to treat conditions such as gastroesophageal reflux. We found that treatment of animals with omeprazole prior to or after the establishment of infection partially rescued Δ*tlpAD H*. *pylori*’s ability to colonize the stomach (Fig 6), while it did not affect the total number of wild-type bacteria.

We previously reported that in this murine model of infection and in humans the majority of the bacteria reside in the overlying mucus layer. However, a subpopulation of *H*. *pylori* can be found deep in the gastric glands adhered to epithelia cells that make up the mid-glandular proliferative zone [7, 11]. We wondered whether acid sensing would affect not only the overall fitness of the bacteria establishing colonization in the stomach, but also their ability to reach and colonize the epithelial surface of the gastric glands. To investigate gastric gland colonization, we used quantitative 3D confocal microscopy to determine the number of bacteria growing as microcolonies within the gastric glands in the antrum and corpus regions of the stomach. One of the main distinguishing features between antrum and corpus is the presence of acid-secreting parietal cell in the corpus glands.

**Fig 7:**
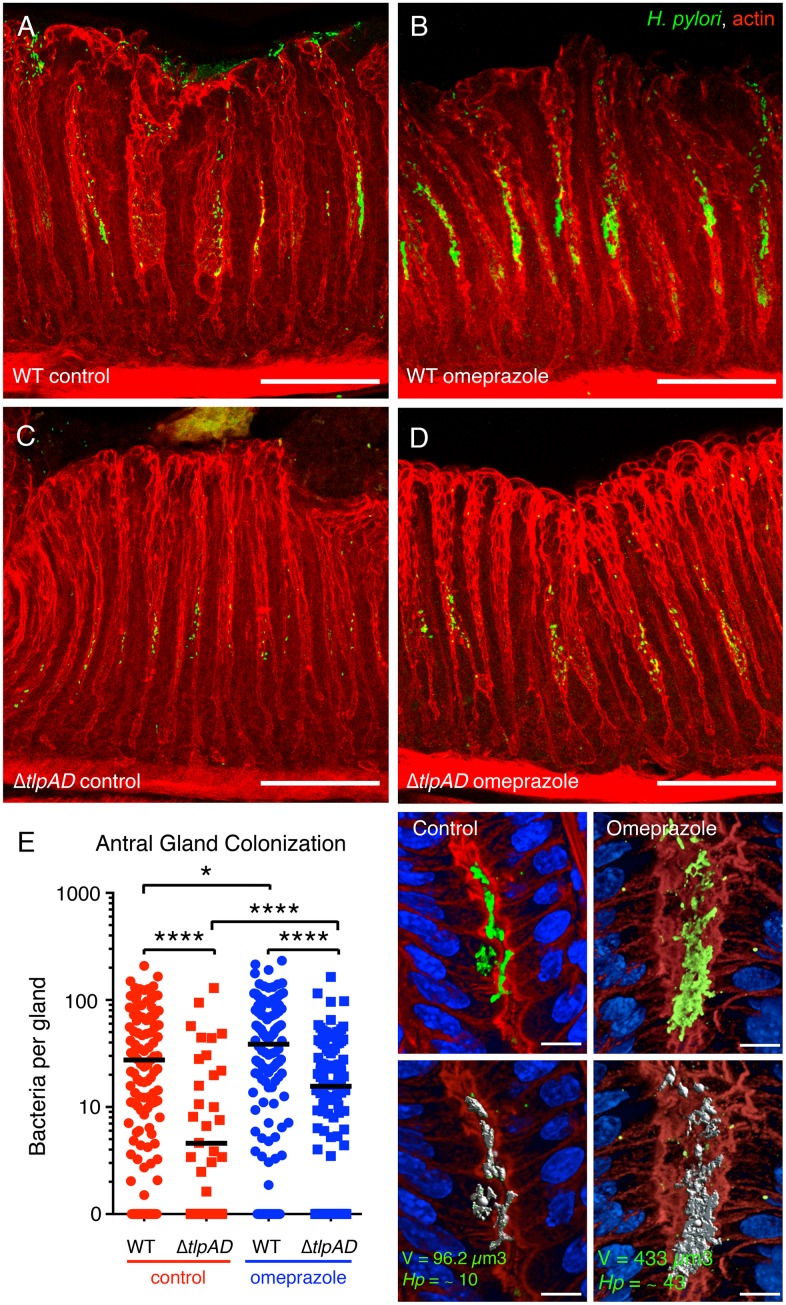
The density of gland-associated *H*. *pylori* in the antrum of the stomach is influenced by gastric acidity. (**A-D**) 3D confocal immunofluorescence reconstructions of mice infected with wild-type (WT) or Δ*tlpAD H*. *pylori* followed by treatment with omeprazole or no treatment. *H*. *pylori* are stained in green and actin in red. Scale bar is 100μm for all 4 panels. (**E**) Quantification of the number of bacteria per gland in the antrum of animals infected with WT or Δ*tlpAD* treated with omeprazole or untreated. The antral glands of seven animals in each cohort were analyzed for bacterial colonization by 3D volumetric analysis. The number of bacteria in each individual gland is plotted as a scatter point. * *P* < 0.05, **** *P* < 0.0001 (Mann Whitney test). The panels on the right show an example of the volumetric measurement of bacteria in gastric glands. 3D confocal reconstructions of glands stained with anti–*H pylori* (*green*), phalloidin (*red*), and DAPI (*blue*) were generated. Bacterial microcolony signals are identified by fluorescence intensity and size using Volocity software and the identified voxels marked in *gray* (*bottom panels*) are measured. The number of bacteria per gland is calculated based on the average voxel volume of 1 bacterium. Scale bar is 10μm for all 4 panels.

In control animals with normal acid secretion, the wild-type strain is found mostly colonizing the antral glands and the transition zone between the antrum and corpus [7, 11] (S14A and S14B Fig) rather than the corpus glands (S14C and S14D Fig). We found that in only two out of seven animals infected with Δ*tlpAD H*. *pylori*, the mutant was able to colonize the antral glands but at significantly lower densities than wild-type *H*. *pylori* (Fig 7A, 7C and 7E). This result suggests that chemotaxis through TlpB and TlpC still allows some bacteria to reach the epithelium and colonize the glands. We know that one such signal is the chemoattractant urea sensed through TlpB [15]. The defect in gland colonization of the mutant, however, may be attributed to low bacterial numbers in both the mucus and the glands as it has a 100-fold defect in overall bacterial load compared to wild-type *H*. *pylori* (Fig 6).

We performed a similar analysis of gastric gland colonization of Δ*tlpAD H*. *pylori* in animals treated with omeprazole after infection, since in these conditions the bacterial load in the stomach was comparable to that of wild-type (Fig 6). When we analyzed the antral glands of infected animals treated with omeprazole one week post-infection, we noted that the bacterial density of both wild-type and Δ*tlpAD H*. *pylori* in the antral glands increased significantly compared to those in control animals (Fig 7B, 7D and 7E). Despite the increase in gland colonization, Δ*tlpAD H*. *pylori* does not reach the levels of wild-type in the antral glands of omeprazole-treated animals (Fig 7E). These results suggest that omeprazole promotes the colonization of *H*. *pylori* in the gastric glands of the antrum, and chemotaxis through TlpA and TlpD is important for proper colonization of the antral glands.

**Fig 8:**
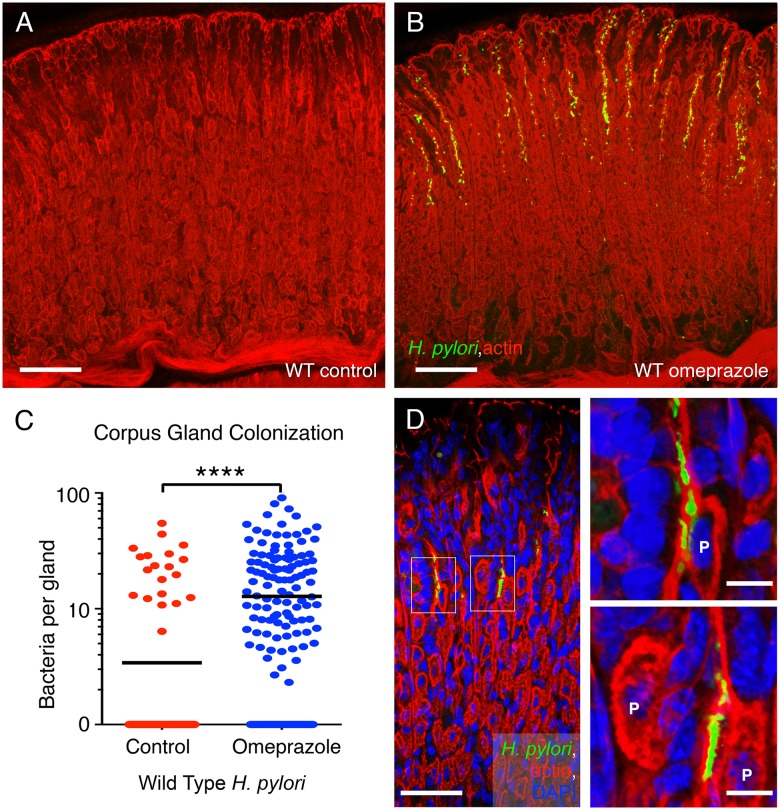
Omeprazole treatment expands *H*. *pylori* colonization into the corpus glands of stomach. (**A-B**) Representative confocal 3D images of the corpus glands infected with wild-type (WT) *H*. *pylori* followed by 1-week treatment or no treatment with omeprazole. *H*. *pylori* are stained in green and the cellular actin cytoskeleton is stained with phalloidin in red. Scale bar 100μm for both panels. (**C**) Quantification of the number of bacteria per gland in the corpus of animals infected with WT *H*. *pylori* treated with omeprazole or untreated control. The corpus glands of seven animals in each cohort were analyzed for bacterial colonization by 3D volumetric analysis. Only one out of seven control animals had detectable bacteria in the corpus. **** *P* < 0.0001 (Mann Whitney test). (**D**) Higher magnification image of the corpus glands from omeprazole-treated animals infected with wild-type *H*. *pylori*. *H*. *pylori* are stained in green and actin in red. Gland-associated *H*. *pylori* are seen in proximity to parietal cells (P) magnified in the insets. *H*. *pylori* are stained with antibodies in green. The glands are visualized with actin staining with phalloidin in red and nuclei are stained with DAPI in blue. Scale bar is 100μm in D and 10μm in the insets in D.

Using quantitative 3D-confocal microscopy, we also found that loss of stomach acidity through omeprazole treatment allowed *H*. *pylori* to extend its colonization to the corpus glands (Fig 8). Control animals normally do not have *H*. *pylori* in corpus glands (Fig 8A). In our analysis, only one out of the seven control animals had detectable levels of *H*. *pylori* in the corpus glands (Fig 8C). In the corpus, the gland-associated bacteria seen after omeprazole treatment were mainly concentrated in the neck region of the glands in the proliferative zone (Fig 8B), and also were seen in close proximity to parietal cells (Fig 8D). This result suggests that the acid in the stomach restricts *H*. *pylori* gland-colonization to the antral glands but that an increase in gastric pH allows *H*. *pylori* to extend its range to also colonize the epithelium of the corpus glands. We did not find Δ*tlpAD H*. *pylori* in the corpus glands of omeprazole-treated animals. These results from the omeprazole treatment experiments suggest that gland colonization is distinct from colonization of the mucus, and sensing through TlpA and TlpD may be necessary for localizing to and/or persisting in the corpus glands.

## Discussion

Our study has revealed that *H*. *pylori* evolved two independent chemoreceptors, TlpA and TlpD, capable of sensing and rapidly responding to acid gradients. The fact that *H*. *pylori* devotes at least half of its chemoreceptor repertoire towards acid sensing underscores the importance of this function for *H*. *pylori’s* survival in the stomach.

Despite the same overall function, we found that there is a difference in the sensitivity of TlpA versus TlpD in detecting HCl. The cytoplasmic TlpD chemoreceptor appears to be more sensitive than the periplasmic TlpA chemoreceptor, and it is able to sense both lower pH as a repellent and higher pH as an attractant. *H*. *pylori* may have evolved a more sensitive acid sensor in its cytoplasm as it would be crucial to detect even small changes in cytoplasmic pH to ensure homeostasis.

TlpD is the only chemoreceptor of the four that *H*. *pylori* possesses that is thought to be a soluble protein located in the bacterial cytoplasm and inner membrane [17]. It has been reported to be an energy sensor causing *H*. *pylori* to repel from electron transport inhibitors and other low energy-inducing environments [17]. Currently it is not known what are TlpD’s specific ligands and how TlpD may be detecting these ligands. TlpD contains a C-terminal chemoreceptor zinc binding domain (CZB) of unknown function [21] and does not contain a PAS domain commonly found in other chemoreceptors, making it challenging to identify the ligands that TlpD directly bind. It is possible that changes in intracellular pH may affect intracellular metabolism and the energy state of the bacterium, which would link TlpD’s ability to sense acid to its role as an “energy sensor,” and consequently, an indirect sensor of acidic pH. Interestingly, it was recently published that changes to particular protein interactions with TlpD or the metabolic state of the bacterium alters the localization of TlpD in *H*. *pylori* [19]. This and another study, also reported that the sensing mechanism of TlpD may be linked to oxidative stress and iron limitation [18, 19]. Specifically, upon metabolic stress or iron limitation, TlpD changes its localization from the poles of the bacterium to the cytoplasm. Also, in the absence of the interacting partners recently identified, TlpD changes its localization from the poles to the cytoplasm [19]. It is unclear, however, how TlpD’s localization to the poles or the cytoplasm affects its ability to sense and respond to particular chemoeffectors. There is no direct evidence that TlpD directly interacts or forms chemoreceptor arrays with TlpA, TlpB or TlpC to signal, and in fact TlpD has been shown to localize to the poles and be capable of transducing a chemotactic signal to the flagellar machinery in the absence of all other chemoreceptors [22]. Structural analysis of the chemoreceptor may reveal more insights into the mechanism of sensing through TlpD.

TlpA has been reported to sense arginine and bicarbonate as attractants in *H*. *pylori* strains 26695 [16] and 700392 [23], but its mechanism of sensing has not been further characterized. It has been proposed that chemotaxis towards a bicarbonate gradient in vivo may help *H*. *pylori* navigate towards a safe niche on the gastric epithelium where it is protected against the acid in the lumen. It is unclear whether TlpA is sensing bicarbonate directly or the basic pH that bicarbonate creates. While our studies here have shown that *H*. *pylori* is attracted to gradients of basic pH, we identify TlpD, not TlpA, as the chemoreceptor necessary for this attraction. It is possible that TlpA may be detecting bicarbonate specifically as a ligand rather than an increase in pH or we did not test the optimal basic pH that TlpA may be detecting. Further studies are needed to determine how TlpA is sensing acidic pH and whether TlpA may be involved in sensing basic pH as well.

Our data surprisingly revealed that loss of TlpB does not result in defects in escaping from an acid gradient in the microgradient assay. However, we noted that the response kinetics of Δ*tlpB H*. *pylori* differed from that of wild-type (Fig 1C). This suggests that lacking TlpB does have an effect on *H*. *pylori’s* ability to respond to acid even though TlpB is neither necessary nor sufficient to detect acid gradients. The difference between these recent results and previously published findings may be attributed to differences in the assays used to assess Δ*tlpB’s* responses to acid.

Our assay generates and maintains a constant microscopic gradient from a point source and records the chemotactic behavior immediately after exposure to a gradient [7, 15]. We observe bacterial responses within seconds, and the response is sustained for long periods of time (we have tested it for as long as 10 minutes). However, our assay does not change the overall pH of the medium containing the bacteria because we inject minute amounts of acid (with a flow rate on the order of picoliters per minute) at very low pressure through a femtotip needle. A previously described assay that places *H*. *pylori* in an acidic environment for several minutes describes the formation of a barrier of bacteria at a region where the pH has been altered [12–14]. In this barrier assay the bacterial culture is infused with a 100 mM solution of HCl, which exceeds the buffering capacity of Brucella broth, and when mixed would decrease the pH of the bacterial culture from about pH 6.7 to pH 4.76, as we determined empirically (S6 Fig). Thus, the media is likely acidified when the chemotactic behavior is observed at about 5 minutes after exposure.

Another assay used that has implicated TlpB’s role in acid sensing is a video chemotaxis assay where the bacteria are placed in chemical solutions of interest, such as acidic solutions [13, 14, 17, 24]. In this assay, the bacteria are not exposed to a chemical gradient, but the assay measures motility behavioral differences in the presence or absence of a chemical as stops/sec or reversals/sec. An increase in stops or reversals per second indicates the detection of a repellent, but since a gradient is absent, directed movement cannot be assayed. An increase in reversals may therefore also represent the loss of an attractant or a change in the functioning of the chemosensory signal transduction pathway. The conditions in these two assays differ drastically from that of the microgradient assay with regard to the shape and steepness of the acid gradient as well as the time scale in which chemotactic responses are assessed. These other two assays may better assess *H*. *pylori’s* response when immersed in a more homogenous low pH environment such as the stomach lumen as opposed to an environment where there is a steep acidic pH gradient, such as across the gastric mucus layer. Perhaps TlpB is important for acid sensing in the acidic lumen.

We do detect differences in the speed and sensitivity of acid sensing by the other receptors when TlpB is missing. Perhaps TlpB plays a role in acid sensing, for example, by changing the sensitivity of acid sensors at different baseline pH conditions or in other spatial and temporal conditions not replicated in the microgradient assay. The altered response kinetics of Δ*tlpB* compared to wild-type may be the resultant response from the integration of the remaining three receptors. Lacking TlpB may be changing the way the other receptors function in sensing acid as well as how other signals present in their environment alter responses to acid. While there is no evidence that TlpB and TlpD directly interact, chemoreceptors are known to form mixed arrays that transduce signaling responses to the flagellar motor [25, 26]. It is possible that TlpB may directly interact with TlpD under certain conditions to dampen its response to basic pH. One intriguing speculation is that sensation and responses to urea through TlpB may also be coupled to sensing cytoplasmic pH through TlpD, through a yet unknown mechanism. Our observation that lacking TlpB enhances *H*. *pylori’s* sensitivity to detecting higher pH suggests this possibility.

Our experiments elucidating acid sensing were all performed under conditions where urea was absent in the culture medium. Urea was only present when we deliberately introduced it in solution with HCl in the needle to investigate *H*. *pylori’s* response to multiple signals. This allowed us to pinpoint specifically the acid-sensing functions of the chemoreceptors. However, in vivo, *H*. *pylori* is exposed to both HCl and urea. While urea is a potent chemoattractant sensed through TlpB, which may have an effect on pH sensing, it is also the substrate for *H*. *pylori’s* highly expressed urease enzyme, whose activity certainly affects pH sensing. As a neutrophile, urease buffering activity is essential for *H*. *pylori’s* survival in vivo. In vitro studies have shown that *H*. *pylori* is able to survive under pH 1 conditions for several hours if the bacteria are in the presence of urea [27]. Many studies have been conducted to elucidate the intricate mechanism of how acidic conditions in the environment trigger a cascade of events resulting in an increase in cytoplasmic pH while the external pH remains acidic. Upon exposure to acidic pH conditions, urease assembles into a complex with a proton-gated urea channel embedded in the inner membrane [28–30]. Urea enters through this proton-gated channel to reach cytoplasmic urease where it optimally functions to degrade urea into ammonia and bicarbonate thereby raising the pH [31].

Based on the findings in these prior studies, we predict that in the presence of urea and urease *H*. *pylori* will be less responsive to an acid gradient in our microgradient assay. Future studies will need to integrate the role of urease and urea into acid sensing since it alters the intracellular and extracellular pH. It would be interesting to determine how urease activity might affect the sensitivities of TlpA and TlpD to acidic pH. This may explain why TlpD is more sensitive to pH since it detects changes in the cytoplasm.

Our newer data show that TlpA and TlpD are the primary acid sensors in *H*. *pylori* that allow immediate response to HCl gradients and are important for stomach colonization in the presence of gastric acid. We find that a mutant lacking both chemoreceptors has a severe defect in establishing colonization in the murine stomach. It has been previously reported that TlpD is important for *H*. *pylori* survival and proliferation in the antrum [20]. We also found that Δ*tlpD H*. *pylori* has an approximate 10-fold defect in overall colonization of the stomach at two weeks post-infection (Fig 6). This could be due to the loss of chemotaxis towards other signals important for survival in addition to acid sensing. Indeed, we report here that TlpD also mediates attraction to environments of higher pH. However, TlpD does contribute to acid sensing *in vivo*, since deletion of TlpA has no effect on colonization *in vivo*, yet double deletion of TlpA and TlpD markedly worsens the colonization defect of the Δ*tlpD H*. *pylori* mutant (Fig 6). Interestingly, it has also been reported that *tlpD* is one of the most upregulated genes when *H*. *pylori* is exposed to acidic conditions in vivo [32]. These results indicate that chemotaxis through TlpD is most crucial for establishing colonization of the stomach, but that TlpA can compensate for the loss of acid-sensing through TlpD.

Furthermore, we discovered that sensing through TlpA and TlpD is important for localizing properly to the gastric glands and that blocking acid secretion with omeprazole changes the distribution of gland-associated *H*. *pylori* in the stomach. We previously reported that bacteria growing as microcolonies in the gastric glands lead to inflammation in the regions of gland colonization. In addition, gland-associated *H*. *pylori* locally activate and induce proliferation of the stem cells in the infected glands leading to hyperplasia [11]. A previous investigation of the effects of omeprazole on *H*. *felis* distribution in the mouse stomach reported that acid suppression extends the colonization range of the bacteria into the corpus glands [33]. A similar finding was reported for mice infected with *H*. *pylori* and treated with omeprazole where the stomach glands were qualitatively found to contain less bacteria in the antrum than in the corpus [34]. Pulsed omeprazole dosing in gerbils has also been shown to alter the orientation of *H*. *pylori* within the mucus layer relative to the gastric epithelium, which could promote bacterial clearance by driving the bacteria closer to the lumen or perhaps improving the efficiency of antibiotics [35]. Our results support these previous findings that reducing gastric acidity through proton pump inhibitor treatment changes bacterial density and distribution in the gastric glands, extending their range within this niche. Our results further suggest that targeting chemosensation through TlpA and TlpD would interfere with overall stomach and gland colonization.

*H*. *pylori* infection in humans most often leads to chronic inflammation in the antrum (antral predominant gastritis). People with antral gastritis usually have no symptoms but may develop pyloric or duodenal ulcers as a consequence of the infection. People at risk for gastric adenocarcinoma are different in that they develop an anatomical pattern of gastritis that extends into the corpus. Corpus gastritis is associated with the loss of parietal cells, low acid secretion, high gastrin production and gastric atrophy (multifocal atrophic gastritis) [36]. These differences in the anatomical localization of the inflammatory changes in *H*. *pylori* infection has been ascribed to physiological differences in individuals, but could also reflect the anatomical site of infection of gland-associated *H*. *pylori*. That is, extension of the distribution of gland-associated bacteria from the antrum to the corpus glands may precede and contribute to the inflammatory and hyperplastic changes that lead to tissue pathology [11].

In our experimental system, gastric colonization of the corpus is promoted by proton pump inhibitor (PPI) treatment, suggesting that this could be a precursor step towards the development of multifocal atrophic gastritis and pre-neoplasia. Experiments in Mongolian gerbils, for example, have shown that PPI treatment can promote the development of adenocarcinoma [37]. In humans, the role of PPIs in atrophic gastritis remains controversial, but it is recommended that patients considered for long-term PPI therapy first be tested and treated for *H*. *pylori* infection [38, 39]. Our study, and others, supports this clinical recommendation and also suggests that targeting pH sensing through TlpA and TlpD may be an effective way of disrupting *H*. *pylori* colonization in the stomach.

## Materials and Methods

### Bacterial strains and culture conditions

The previously published wild-type *H*. *pylori* strain PMSS1 [40], Δ*ureAB* and single knock-outs chemoreceptor mutants [15] were used for all experiments in this study except those strains noted in S4 Fig. *H*. *pylori* strains were either grown on Columbia blood agar plates or in Brucella broth supplemented with 10% fetal bovine serum (BB10) at 37°C, 10% CO_2_, as described previously [41]. The Δ*tlpAB*, Δ*tlpAC*, Δ*tlpAD*, Δ*tlpBC*, Δ*tlpBD*, and Δ*tlpCD* PMSS1 isogenic mutants were constructed by natural transformation with genomic DNA from the relevant single knock-out chemoreceptor mutant made in strain PMSS1. The PMSS1 chemoreceptor mutants were verified by immunoblotting using an antibody that recognizes a conserved domain in all four chemoreceptors, courtesy of K. Ottemann [42].

### Test solutions for microgradient assay

All solutions were diluted or dissolved in sterile, distilled, and deionized water. Urea and sodium hydroxide solutions were made by dissolving ultra-pure urea or sodium hydroxide tablets into sterile, distilled and deionized water. One molar hydrochloric acid (Sigma Aldrich, Saint Louis, MO) was diluted into sterile, distilled and deionized water to create various concentrations of hydrochloric acid solutions to test. To obtain phosphate buffered solutions with different pHs, 1 M monobasic sodium phosphate (NaH_2_PO_4_) pH 4.1 and 0.5 M dibasic sodium phosphate (Na_2_HPO_4_) pH 9.0 were combined in various proportions until desired pH is achieved and confirmed by pH meter. To obtain citrate buffered solutions with different pHs, 1 M citric acid (C_6_H_8_O_7 •_ H_2_O) pH 2.0 and 1 M sodium citrate (C_6_H_5_O_7_Na_3 •_ 2H_2_O) pH 8.6 were combined in various proportions until desired pH is achieved and confirmed by pH meter. The buffering capacity and corresponding pH of various concentrations of HCl in BB10 was determined empirically (S6 Fig).

### Microgradient assay

*H*. *pylori* cultures used for the assay were made by subculturing from a 16-hour overnight Brucella broth + 10% FBS (BB10) culture with a starting OD_600_ of 0.15. The subcultures (also in BB10) were grown for 6 hours until they reach an OD_600_ of 0.3 before using in the microgradient assay. The pH of bacterial cultures prior to the microgradient assay (spent media) was measured at a mean of 6.65 +/- 0.04 SD (S1B Fig). These were not different from the pH of BB10 kept in the incubator without bacteria measured at 6.6, or freshly made BB10 (pH 6.6–6.8). The bacterial culture media for all strains tested, except Δ*ureAB*, contain no detectable urea at the time of the assay due to bacterial urease activity during the subculture (S1A Fig). This was determined by measuring the concentration of urea using the QuantiChrom Urea Assay Kit (BioAssay Systems), a colorimetric assay that quantifies urea directly with a detection level of 13μM. Background was determined by quantifying urea in urease-treated BB10. Twenty five milliliters of fresh BB10 (predicted to contain 2–4 mM urea) were incubated with 300 units of Jack Bean urease for 2 hours at 25°C. This amount of urease is calculated to liberate 0.3 millimole of ammonia in 1 min at 25°C, and therefore is sufficient to hydrolyze all the urea in the media in about one minute. To ensure complete urea degradation, we allowed the reaction to proceed for 2 hours before boiling the urease-treated media for 10 minutes to inactivate urease and filtering the media through a 0.2 μm filter to remove particulates.

Two hundred seventy microliters of the subculture were placed into the center of a glass-bottom 35-mm dish (MatTek) contained by a ring of vacuum grease. The dish was placed above a 32x objective of a Zeiss Axiovert-35 inverted microscope equipped with phase-contrast optics and a heated stage (37°C). A Hammamatsu C2400 video charge-coupled-device (CCD) camera was used to record via an Argus-20 image processor onto Quicktime at 30 frames per second. A Femtotip II microinjection micropipette (Eppendorf) containing 8 μl of the test solution was inserted into or removed from the viewing field using a micromanipulator (Eppendorf 5171) [7]. To create a microscopic gradient (microgradient) in the bacterial culture, a compensation pressure of 30 hPa was applied via the Eppendorf transjector 5246 to maintain a constant flow at 0.372 picoliters/minute from the tip. This pressure was determined empirically and selected because it did not physically affect the bacteria swimming near the micropipette tip while generating a stable gradient within the viewing field.

### Quantification of bacterial chemotactic responses to microgradients and display of bacterial traces

*H*. *pylori* chemotactic responses were quantified from phase contrast video microscopy movies recorded at 30 frames per second using ImageJ software version 1.46r. Movies were analyzed starting from 4 to 10 seconds before micropipette insertion (pre-injection) to 10–30 seconds after micropipette insertion (post-injection). After background subtraction and contrast adjustment, movies were then subsequently quantified or bacterial traces were obtained for visual depiction of swimming behavior in response to the gradient. For quantification, 0.2 second segments of the movies (6 frames) were combined into Z-projections to generate traces of moving bacteria. Motile bacteria were detected by specifying a size between fifty to three hundred pixels and circularity values between 0.1 to 0.5 (non-motile particles will be more circular than motile bacteria, which will be elliptical traces). These parameters were set in the Analyze Particle function in ImageJ and the output provided the number of motile bacteria per frame. The number of motile bacteria per frame is normalized to the average number of motile bacteria prior to needle entry. Responses are shown as either scatter plots, which display the percent bacteria per field from 4–5 seconds pre-injection to 10–15 seconds post-injection, or bar graphs, which display the percent bacteria per field at 4 second pre-injection and 10 seconds post-injection. Statistical significance between the percent bacteria per field pre-injection versus percent bacteria per field post-injection in the bar graphs or of the overall response in the scatter plots was assessed using a 2-way repeated measures ANOVA. To produce the images that depict the bacterial swimming behavior, longer traces were generated to clearly show the response. The time stated in the pre-injection panel indicates the first frame until 0 seconds used to generate the trace (i.e. -1.5 sec means frames from 1.5 seconds prior to needle entry to 0 second when the needle enters were used to generate a 1.5 trace representing the pre-injection swimming behavior). The range of time stated in the post-injection panels indicates the frames used to generate the trace (i.e. 18.5–20 sec means the frames from 18.5 seconds to 20 seconds post-injection were used to generate a 1.5 trace representing the post-injection swimming behavior).

### Immunoblotting

*H*. *pylori* cells were harvested from blood plates and lysed with 1x SDS sample buffer. Lysates were boiled for 5 minutes and then separated on a 10% SDS-PAGE gel. After transfer onto nitrocellulose membranes, *H*. *pylori* TlpA, TlpB, TlpC, TlpD were detected by blotting with rabbit anti-tlpA22, an antibody that recognizes a conserved domain in all 4 chemoreceptors, courtesy of K. Ottemann [42], followed by a goat anti-rabbit Alexa Fluor 660. UreA, the small subunit of the abundantly expressed urease protein, was used as a loading control and detected with a mouse anti-UreA antibody followed by a goat anti-mouse Alexa Fluor 800. The blot was then scanned with a Licor-Odyssey scanner at 700 and 800 nanometers.

### Ethics statement

Experiments involving animals were performed in accordance with NIH guidelines, the Animal Welfare Act, and US federal law. All animal experiments were approved by the Stanford University Administrative Panel on Laboratory Animal Care (APLAC) and overseen by the Institutional Animal Care and Use Committee (IACUC) under Protocol ID 9677. Animals were euthanized by CO_2_ asphyxiation followed by cervical dislocation.

### Animal experiments

6 week-old female C57BL/6J mice were purchased from the Jackson Laboratory (Bar Harbor, ME). Animals were infected intraorally by allowing the animals to drink a 5 microliter suspension containing 10^8^ CFU of *H*. *pylori* grown in BB10 from a pipette tip [7]. One cohort of animals was treated with omeprazole at 400 μmol/kg/day via drinking water for seven days after seven days of infection. A second cohort of animals was treated with omeprazole (400 μmol/kg/day) for three days prior to infection and then maintained on omeprazole for the duration of the two-week infection. The control cohort was given untreated water throughout the course of the two week infection. Animals were sacrificed at 2 weeks post-infection by CO_2_ asphyxiation. The stomach was harvested with the forestomach removed and discarded, opened via the lesser curvature, and laid flat onto filter paper. Luminal content was removed and the stomach was divided into halves that spanned the corpus to the antrum. One half of the stomach was weighed and mechanically homogenized for 30 seconds in 1 ml Brucella broth. Homogenized stomachs were serially diluted and plated for CFU counts. The data represent the number of CFU/gram of stomach. Bars represent the geometric mean. Statistical significance in the recovered bacterial load between strains was assessed by a Mann Whitney test. The efficacy of the omeprazole treatment was determined via a separate experiment where 5 mice were administered omeprazole in their drinking water for 3 days prior to sacrifice and 5 mice were given water for 3 days prior to sacrifice (S13 Fig). To measure the pH of the stomach, the stomach was cut along the lesser curvature and splayed open. The stomach tissue along with its contents were placed at the bottom of a test tube (with opening wide enough to fit a pH probe) with the stomach lumen facing up. One milliliter of sterile water was added to the stomach in the tube. The pH was then measured using a pH meter.

### Immunofluorescence imaging of stomach sections

The other half of the stomach was fixed in a 2% paraformaldehyde and stained for confocal microscopy as previously described [7, 11]. A custom made rabbit anti-*H*. *pylori* PMSS1 antibody and chicken anti-rabbit Alexa Fluor 488 antibody was used. DAPI (4 =, 6-diamidino-2-phenylindole) and Alexa Fluor 594-phalloidin (Molecular Probes) were used for visualization of the nuclei and actin cytoskeleton. Samples were imaged with a Zeiss LSM 700 confocal microscope and z-stacks were reconstructed into 3D images using Volocity software (Improvision). Number of bacteria per gland was determined with measurement functions in Volocity.

### Statistical analyses

All microgradient assay data comparing percent bacteria per field at 4 seconds pre-injection to percent bacteria per field at 10 seconds post-injection as well as the scatter plots of percent bacteria per field over time were analyzed via a 2-way repeated measures ANOVA test in the GraphPad Prism 7 software program. The p-value for the scatter plots indicates the significance of the time by group interaction via a 2-way repeated measures ANOVA. For animal experiments, statistical significance was assessed via a Mann Whitney test. Center values are geometric means and error bars represent standard deviation (s.d.). *n* indicates the number of movies per condition or the number of animals used. NS indicates no statistical significance, * *P* < 0.05, ** *P* < 0.01, *** *P* < 0.001, **** *P* < 0.0001.

## Supporting Information

S1 Fig Wild-type and chemoreceptor mutant culture media are depleted of urea and have a pH of about 6.7. (**A**) Urea concentration (millimolar) of fresh Brucella broth with 10% fetal bovine serum (BB10), spent media from 2 hour or 6 hour subcultures of PMSS1 strains wild-type, Δ*ureAB*, or chemoreceptor mutants. (**B**) pH of media assayed for urea concentration in (A): fresh BB10, spent media from 6 hour subcultures of wild-type, chemoreceptor mutants, and Δ*ureAB*. NS indicates no statistical significance, **** *P* < 0.0001 (Tukey’s multiple comparisons test).
(TIF)

S2 Fig Wild-type PMSS1’s response to opposing chemoeffectors depends on the magnitudes of the chemoeffectors. Still images of bacterial motility traces (lasting 2 seconds) of wild-type PMSS1 before (panels in left column) and after exposure to a mixture of 5 mM urea plus 10 mM HCl, 20 mM HCl, or 50 mM HCl (panels in right column). The positions of the needle tips are marked in yellow.
(TIF)

S3 Fig *H*. *pylori’s* urease is not required for pH sensing and exogenous urea in the culture medium does not affect *H*. *pylori’s* response to hydrochloric acid. **(A**) Still images of bacterial motility traces (lasting 2 seconds) of Δ*ureAB* PMSS1 and Δ*tlpB* PMSS1 before (panels in left column) and after exposure to a mixture of 5 mM urea plus 50 mM HCl (panels in right column). The positions of the needle tips are marked in yellow. (**B**) Quantification of the responses of Δ*ureAB* PMSS1 vs WT PMSS1 to a 100 mM hydrochloric acid gradient. Each point represents the percent of swimming bacteria remaining in the field of view at each time point in the digitized video microscopy movie frames. Points for three representative movies are plotted per strain. Time zero is defined as the moment the needle is introduced and the gradient is initiated.
(TIF)

S4 Fig Δ*tlpB* of different strains of *H*. *pylori* respond to hydrochloric acid. Quantification of the responses of Δ*tlpB* of *H*. *pylori* strains 7.13, G27, SS1, and PMSS1 (second independent clone) to a 100 mM hydrochloric acid gradient. Each point represents the percent of swimming bacteria remaining in the field of view at each time point in the digitized video microscopy movie frames. Points for one representative movie are plotted per strain. Time zero is defined as the moment the needle is introduced and the gradient is initiated.
(TIF)

S5 Fig *H*. *pylori* responds to sulfuric acid and phosphoric acid through TlpA and TlpD. (**A**) Quantification of the responses of wild-type (WT) *H*. *pylori* vs. Δ*tlpA*, Δ*tlpB*, Δ*tlpD*, Δ*tlpAD* to a 500 mM sulfuric acid gradient. Each point represents the percent of swimming bacteria remaining in the field of view at each time point in the digitized video microscopy movie frames. Points for one representative movie are plotted per strain. Time zero is defined as the moment the needle is introduced and the gradient is initiated. (**B**) Quantification of the responses of WT *H*. *pylori* vs. Δ*tlpA*, Δ*tlpB*, Δ*tlpD*, Δ*tlpAD* to a 100 mM phosphoric acid gradient. Each point represents the percent of swimming bacteria remaining in the field of view at each time point in the digitized video microscopy movie frames. Points for one representative movie are plotted per strain. Time zero is defined as the moment the needle is introduced and the gradient is initiated.
(TIF)

S6 Fig *H*. *pylori* growth media has buffering capacity to acid. The buffering capacity of Brucella Broth with 10% FBS (BB10) was determined by adding known amounts of HCl to a volume of media and measuring the change in pH. The x-axis shows the predicted pH if the same amount of acid was added to water. The y-axis shows the measured pH in BB10 or in spent media (BB10 spent) collected after the chemotaxis experiments and filtered to remove the bacteria. The data from six different sets of media titration curves are presented. The blue line is an approximate best fit curve through the data.
(TIF)

S7 Fig Loss of TlpB does not affect the sensitivities of TlpA and TlpD in acid sensing. Still images of bacterial motility traces (lasting 1.5 seconds) of wild-type *H*. *pylori* vs. Δ*tlpB* vs. Δ*tlpA* vs. Δ*tlpAB* vs. Δ*tlpD* vs. Δ*tlpBD* before (panels in left column) and after exposure to a 15 mM HCl gradient (panels in right column). The positions of the needle tips are marked in yellow.
(TIF)

S8 Fig Urea sensing does not change the sensitivity hierarchy of the TlpA and TlpD acid sensors. Still images of bacterial motility traces (lasting 2 seconds) of wild-type PMSS1, Δ*tlpA* PMSS1, Δ*tlpD* PMSS1, Δ*tlpAD* PMSS1 before (panels in left column) and after exposure to a mixture of 5 mM urea plus 50 mM HCl (panels in right column). The positions of the needle tips are marked in yellow.
(TIF)

S9 Fig *H*. *pylori* repels from acidic pH buffered by citrate buffer. (**A**) Quantification of the responses of WT *H*. *pylori* to pH 4, 5, 6 buffered by citrate buffer. The response of Δ*tlpAD* to pH 4 is also shown. Each point represents the percent of swimming bacteria remaining in the field of view at each time point in the digitized video microscopy movie frames. Points for one representative movie are plotted per strain. Time zero is defined as the moment the needle is introduced and the gradient is initiated. (**B**) Still images of WT or Δ*tlpAD* motility traces lasting 2 seconds pre- and post- injection of citrate buffer at various pHs, from movies quantified in A. The positions of the needle tips are marked in yellow.
(TIF)

S10 Fig Absence of TlpB enhances *H*. *pylori’s* sensitivity to basic changes in pH. Still images of bacterial motility traces (lasting 5 seconds) of wild-type *H*. *pylori* vs. Δ*tlpB* before (panels in first and third columns, respectively) and after injection of phosphate buffer solutions with pH 9.2, 7.25, or 7.0 (panels in second and fourth columns, respectively). The pH of the culture medium was 6.7. The positions of the needle tips are marked in yellow.
(TIF)

S11 Fig *H*. *pylori* is also attracted to sodium hydroxide through TlpD. Still images of bacterial motility traces (lasting 5 seconds) of wild-type *H*. *pylori* vs. Δ*tlpA* vs. Δ*tlpD* before (panels in left column) and after injection of a 40mM sodium hydroxide solution with pH 12.6 (panels in right column). The pH of the culture medium was 6.7. The positions of the needle tips are marked in yellow.
(TIF)

S12 Fig Loss of TlpB does not affect expression levels of TlpA and TlpD in *H*. *pylori*. Immunoblot of *H*. *pylori* chemoreceptors from strain PMSS1. Whole cell SDS-SB lysates of Δ*tlpA*, Δ*tlpB*, Δ*tlpC*, Δ*tlpD*, Δ*tlpAD* and WT *H*. *pylori* were analyzed. An antibody that recognizes a conserved domain in all four chemoreceptors was used. UreA, the smaller subunit of urease, was used as a loading control. The band corresponding to each chemoreceptor and UreA are indicated.
(TIF)

S13 Fig Omeprazole treatment of mice raises the pH in the stomach. Omeprazole was administered in the drinking water as described in the Methods section. Food was not restricted. After 3 days of treatment, five mice were sequentially euthanized with carbon dioxide and the stomach immediately removed, opened through the lesser curvature and placed in 1 ml of distilled water. The stomach contents were mixed with the water and a pH probe inserted for measurements. The pH of stomachs harvested from 5 mice administered omeprazole in their drinking waters for 3 days versus 5 untreated mice are plotted. Significance was assessed by a Mann Whitney test.
(TIF)

S14 Fig Examples of the distribution of bacterial microcolonies in the gastric glands. (**A-B**) 3D confocal immunofluorescence reconstruction of a region of the antrum of a mouse infected with wild-type *H*. *pylori* for two weeks. The boxed area is magnified in (**B**). (**C- D**) Corpus of the same mouse in low (**C**) and high (**D**) magnification. Parietal cells can be recognized by their large size and actin staining of the canalicular invaginations (P). *H*. *pylori* are absent from the corpus glands. *H*. *pylori* are stained with antibodies in green. The glands are visualized with actin staining with phalloidin in red and nuclei are stained with DAPI in blue. Scale bar is 100μm in A, C and 10μm in B, D.
(TIF)

S1 Movie *H*. *pylori* can simultaneously sense repellents and attractants. The responses of live cultures of WT *H*. *pylori* vs. Δ*tlpB H*. *pylori* to a microgradient of 5mM urea and 50mM hydrochloric acid. The microgradient is generated from the micropipette tip that comes into the field from the left. The responses of the bacteria were recorded from 4 seconds pre-injection to 40 seconds post-injection.
(MOV)

S2 Movie Δ*tlpB H*. *pylori* swims away from hydrochloric acid gradients. The responses of live cultures of WT *H*. *pylori* vs. Δ*tlpB H*. *pylori* vs. Δ*cheW H*. *pylori* to a microgradient of 100mM hydrochloric acid. The microgradient is generated from the micropipette tip that comes into the field from the left. The responses of the bacteria were recorded from 4 seconds pre-injection to 10 seconds post-injection.
(MOV)

S3 Movie Δ*tlpAD H*. *pylori* does not respond to hydrochloric acid gradients. The responses of live cultures of WT *H*. *pylori* vs. Δ*tlpAD H*. *pylori* to a microgradient of 100mM hydrochloric acid. The microgradient is generated from the micropipette tip that comes into the field from the left. The responses of the bacteria were recorded from 4 seconds pre-injection to 17.6 seconds post-injection.
(MOV)

S4 Movie *H*. *pylori* is attracted to and repelled by phosphate buffer gradients with pHs above and below the medium pH, respectively. The responses of live cultures of WT *H*. *pylori* to microgradients of 1M phosphate buffer solution with pH 6.5 or pH 7.1 (corresponding to an increase of about 110 nM [H+] or decrease of about -115 nM [H+], respectively, compared to [H+] in the culture medium). The microgradient is generated from the micropipette tip that comes into the field from the left. The responses of the bacteria were recorded from 10 seconds pre-injection to 30 seconds post-injection.
(MOV)

S5 Movie *H*. *pylori’s* attraction to higher pH is mediated through TlpD. The responses of live cultures of WT *H*. *pylori* vs. Δ*tlpD H*. *pylori* to microgradients of 1M phosphate buffer solution with pH 9.2. The microgradient is generated from the micropipette tip that comes into the field from the left. The responses of the bacteria were recorded from 5 seconds pre-injection to 26 seconds post-injection.
(MOV)
