# Supplementary figures and images for: Multiple Acid Sensors Control Helicobacter pylori Colonization of the Stomach
Source: PLoS Pathog. 2017 Jan 19;13(1):e1006118. doi: 10.1371/journal.ppat.1006118 (PMC5245789; doi:10.1371/journal.ppat.1006118)

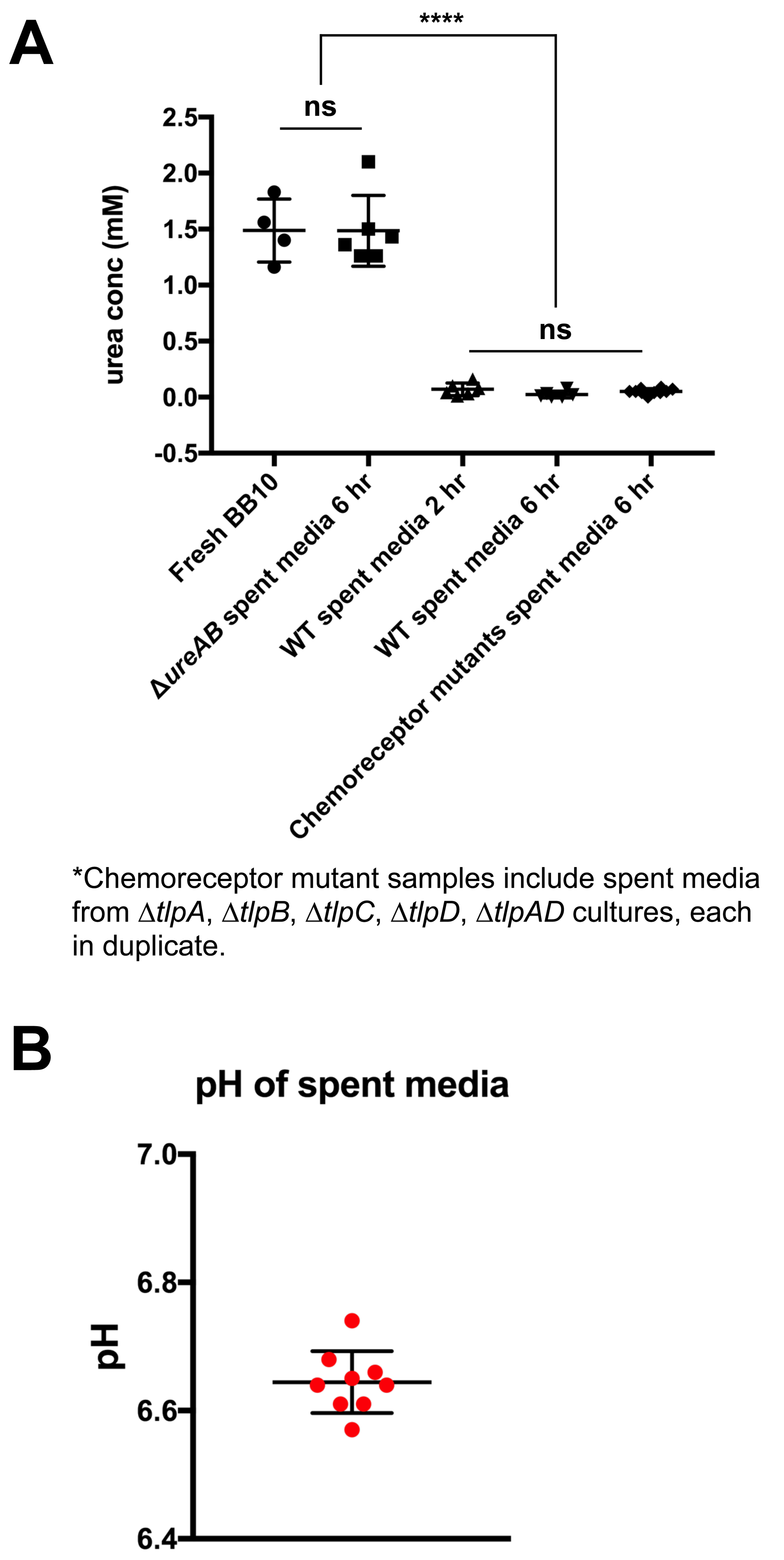

Supplement: S1 Fig — (A) Urea concentration (millimolar) of fresh Brucella broth with 10% fetal bovine serum (BB10), spent media from 2 hour or 6 hour subcultures of PMSS1 strains wild-type, ΔureAB, or chemoreceptor mutants. (B) pH of media assayed for urea concentration in (A): fresh BB10, spent media from 6 hour subcultures of wild-type, chemoreceptor mutants, and ΔureAB. NS indicates no statistical significance, **** P < 0.0001 (Tukey’s multiple comparisons test). (TIF) [file ppat.1006118.s001.tif]

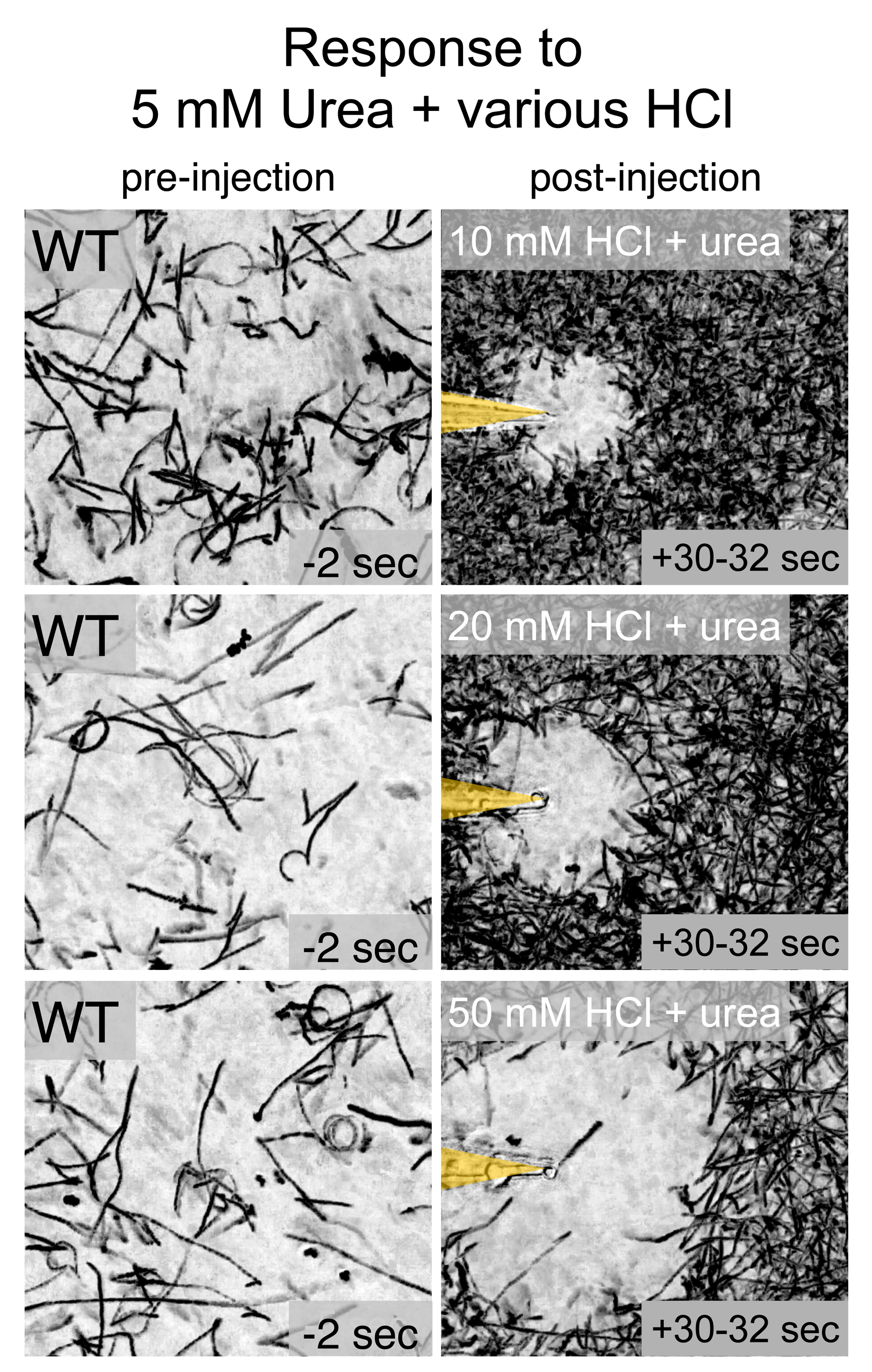

Supplement: S2 Fig — Still images of bacterial motility traces (lasting 2 seconds) of wild-type PMSS1 before (panels in left column) and after exposure to a mixture of 5 mM urea plus 10 mM HCl, 20 mM HCl, or 50 mM HCl (panels in right column). The positions of the needle tips are marked in yellow. (TIF) [file ppat.1006118.s002.tif]

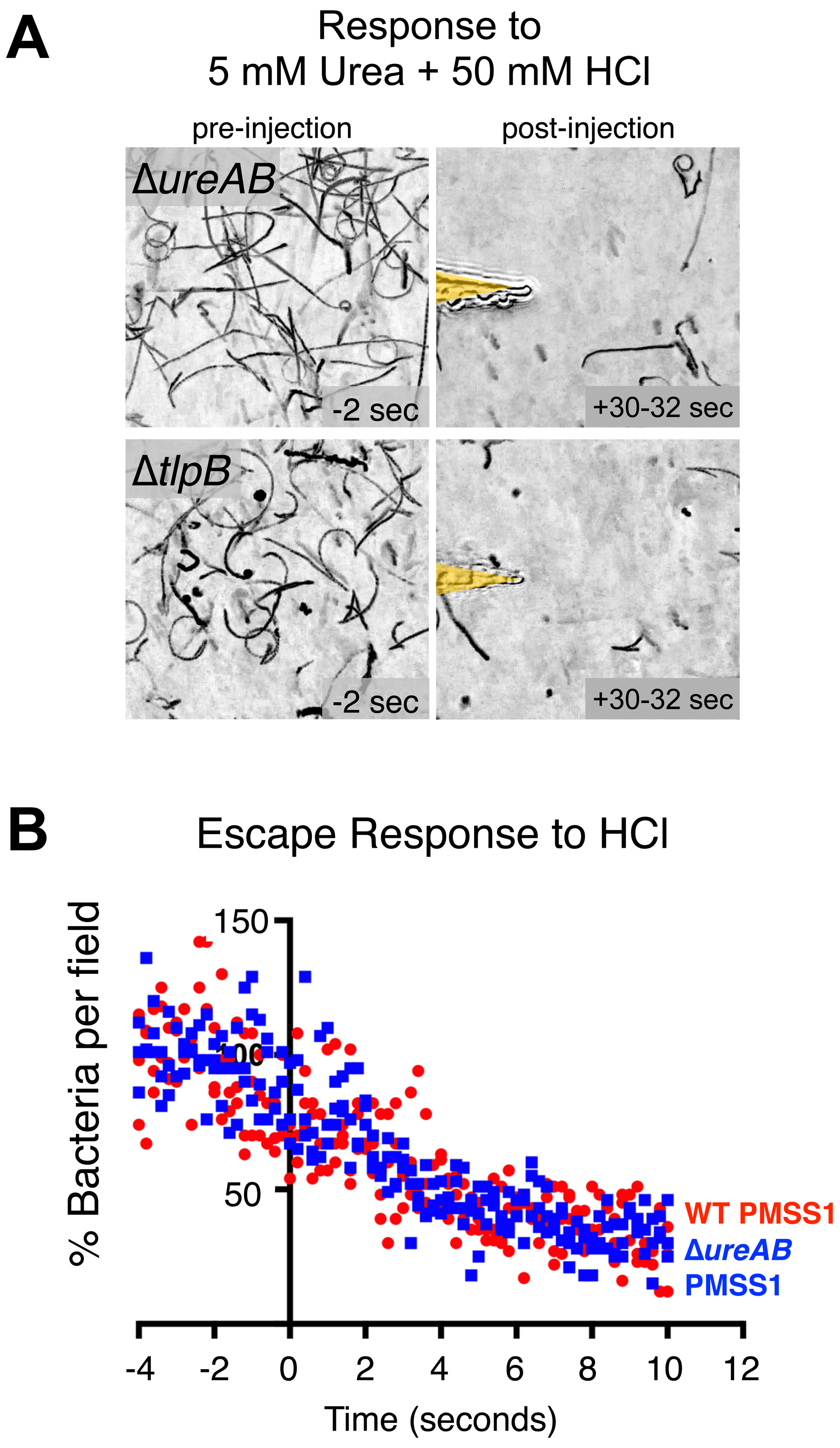

Supplement: S3 Fig — (A) Still images of bacterial motility traces (lasting 2 seconds) of ΔureAB PMSS1 and ΔtlpB PMSS1 before (panels in left column) and after exposure to a mixture of 5 mM urea plus 50 mM HCl (panels in right column). The positions of the needle tips are marked in yellow. (B) Quantification of the responses of ΔureAB PMSS1 vs WT PMSS1 to a 100 mM hydrochloric acid gradient. Each point represents the percent of swimming bacteria remaining in the field of view at each time point in the digitized video microscopy movie frames. Points for three representative movies are plotted per strain. Time zero is defined as the moment the needle is introduced and the gradient is initiated. (TIF) [file ppat.1006118.s003.tif]

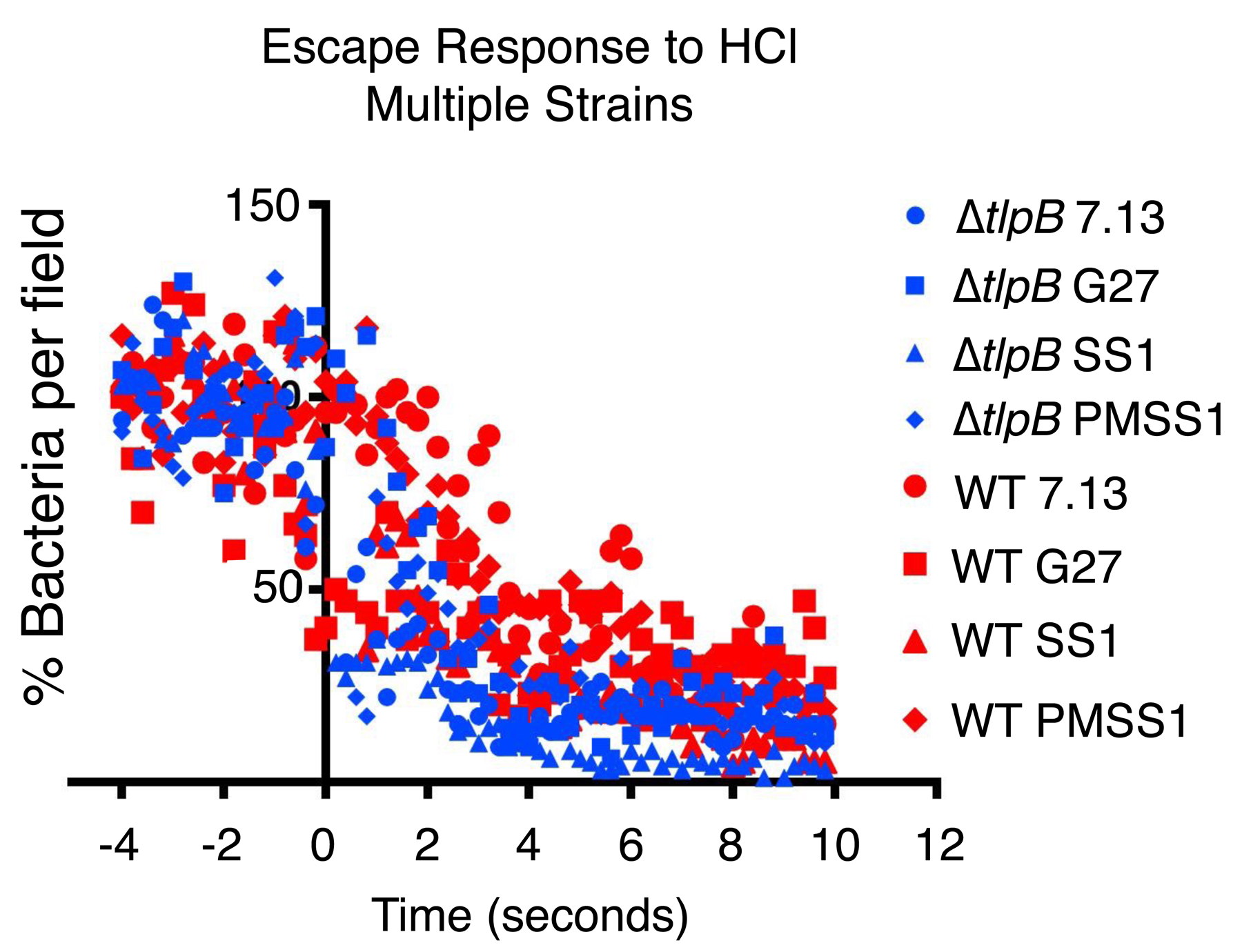

Supplement: S4 Fig — Quantification of the responses of ΔtlpB of H. pylori strains 7.13, G27, SS1, and PMSS1 (second independent clone) to a 100 mM hydrochloric acid gradient. Each point represents the percent of swimming bacteria remaining in the field of view at each time point in the digitized video microscopy movie frames. Points for one representative movie are plotted per strain. Time zero is defined as the moment the needle is introduced and the gradient is initiated. (TIF) [file ppat.1006118.s004.tif]

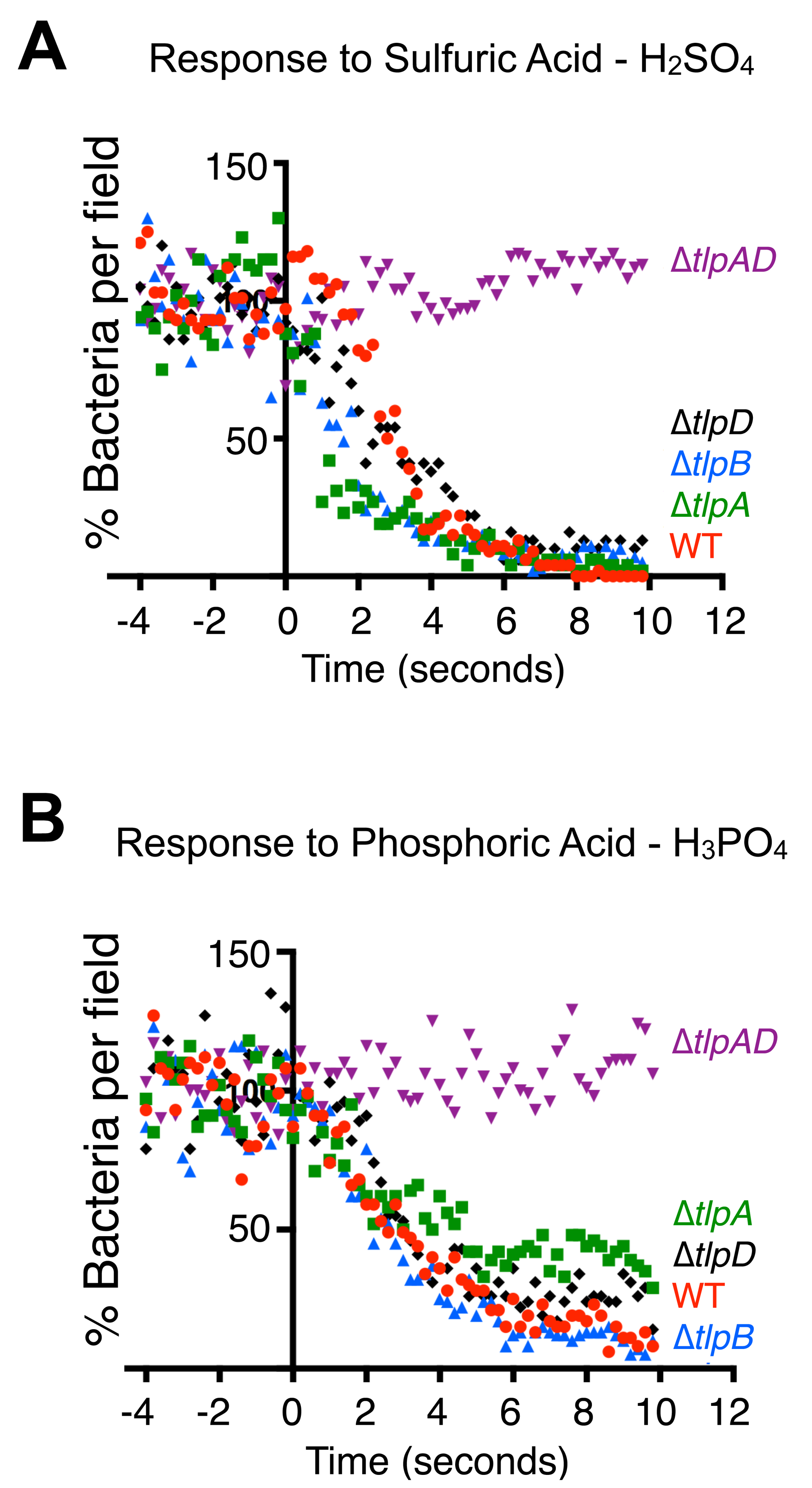

Supplement: S5 Fig — (A) Quantification of the responses of wild-type (WT) H. pylori vs. ΔtlpA, ΔtlpB, ΔtlpD, ΔtlpAD to a 500 mM sulfuric acid gradient. Each point represents the percent of swimming bacteria remaining in the field of view at each time point in the digitized video microscopy movie frames. Points for one representative movie are plotted per strain. Time zero is defined as the moment the needle is introduced and the gradient is initiated. (B) Quantification of the responses of WT H. pylori vs. ΔtlpA, ΔtlpB, ΔtlpD, ΔtlpAD to a 100 mM phosphoric acid gradient. Each point represents the percent of swimming bacteria remaining in the field of view at each time point in the digitized video microscopy movie frames. Points for one representative movie are plotted per strain. Time zero is defined as the moment the needle is introduced and the gradient is initiated. (TIF) [file ppat.1006118.s005.tif]

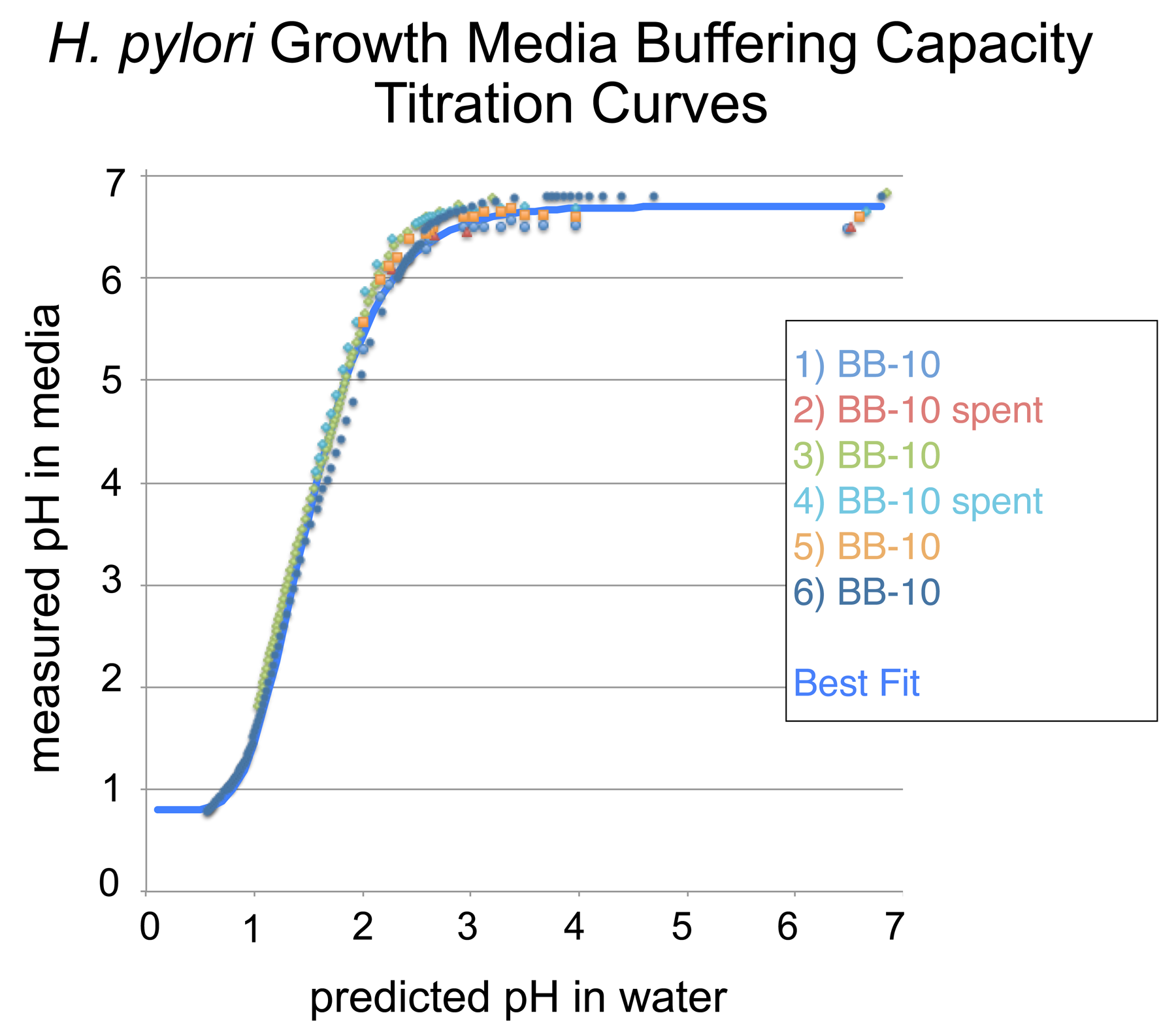

Supplement: S6 Fig — The buffering capacity of Brucella Broth with 10% FBS (BB10) was determined by adding known amounts of HCl to a volume of media and measuring the change in pH. The x-axis shows the predicted pH if the same amount of acid was added to water. The y-axis shows the measured pH in BB10 or in spent media (BB10 spent) collected after the chemotaxis experiments and filtered to remove the bacteria. The data from six different sets of media titration curves are presented. The blue line is an approximate best fit curve through the data. (TIF) [file ppat.1006118.s006.tif]

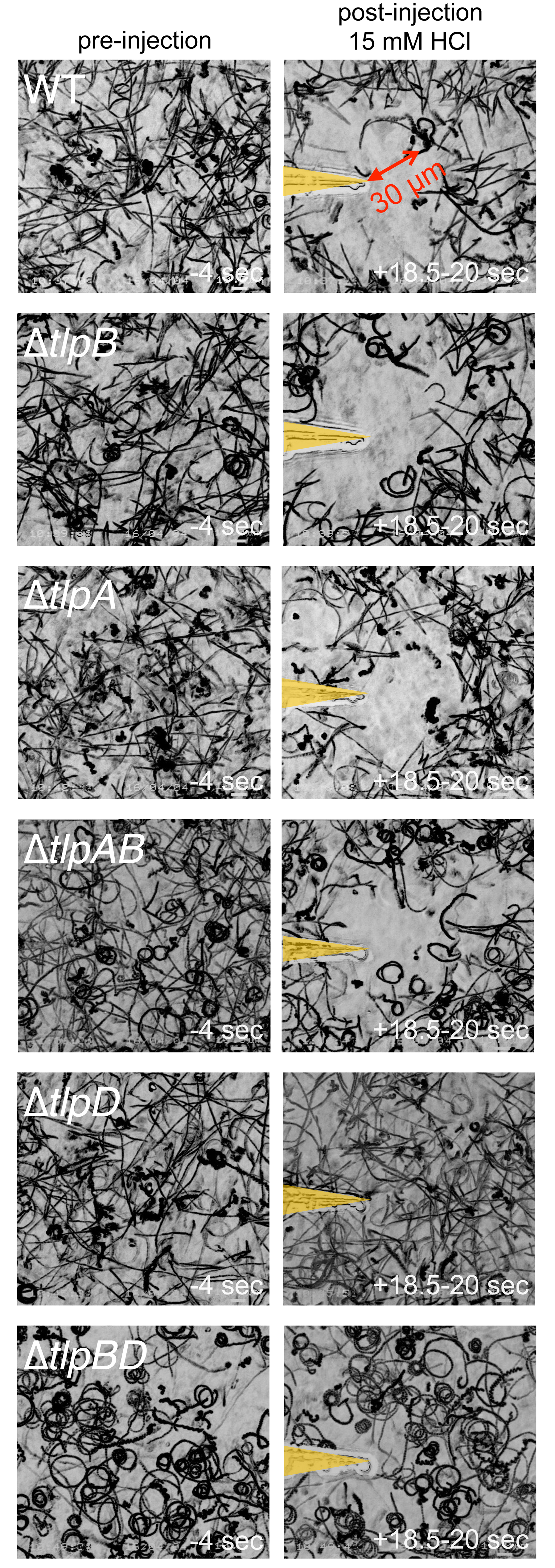

Supplement: S7 Fig — Still images of bacterial motility traces (lasting 1.5 seconds) of wild-type H. pylori vs. ΔtlpB vs. ΔtlpA vs. ΔtlpAB vs. ΔtlpD vs. ΔtlpBD before (panels in left column) and after exposure to a 15 mM HCl gradient (panels in right column). The positions of the needle tips are marked in yellow. (TIF) [file ppat.1006118.s007.tif]

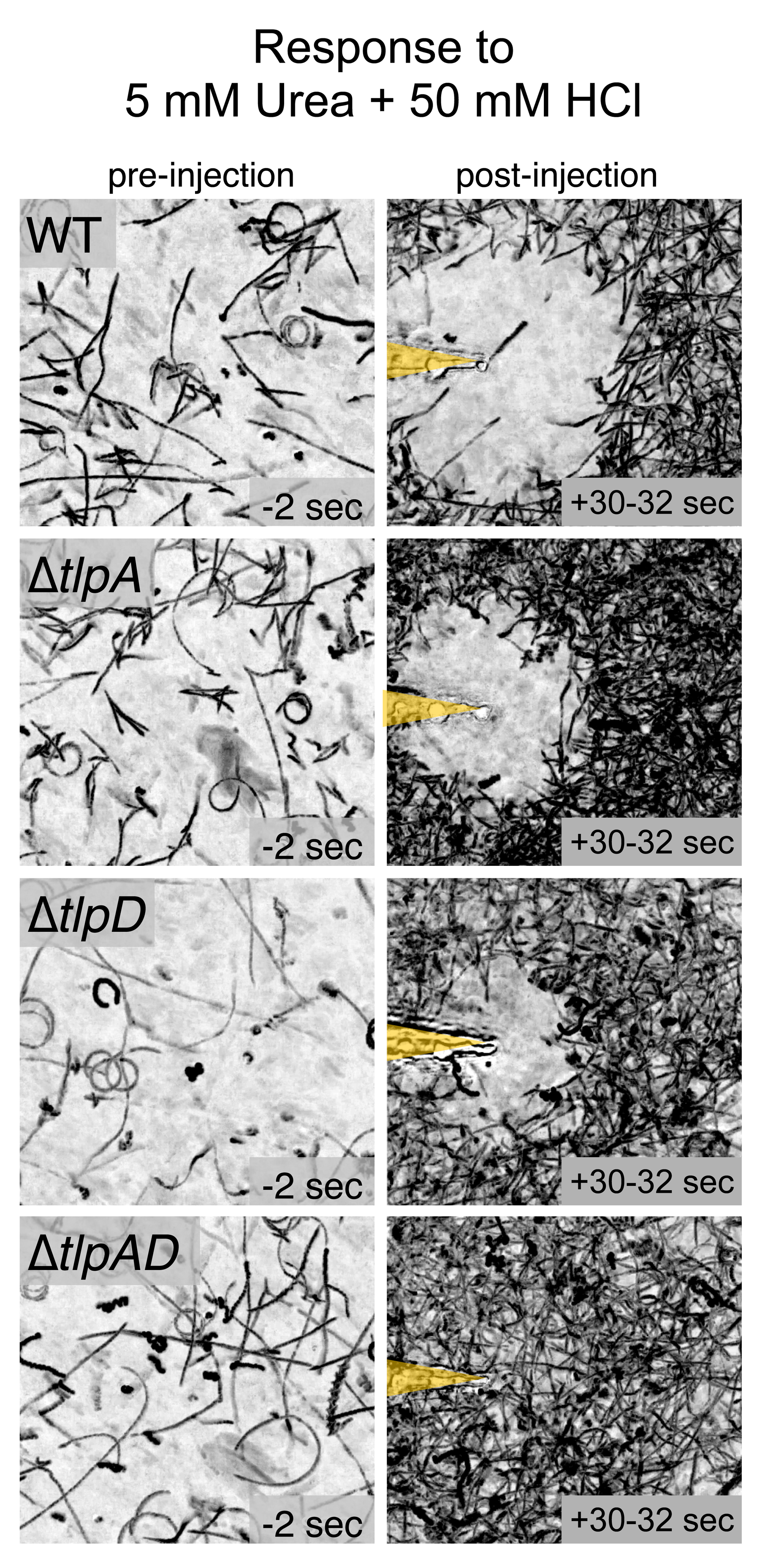

Supplement: S8 Fig — Still images of bacterial motility traces (lasting 2 seconds) of wild-type PMSS1, ΔtlpA PMSS1, ΔtlpD PMSS1, ΔtlpAD PMSS1 before (panels in left column) and after exposure to a mixture of 5 mM urea plus 50 mM HCl (panels in right column). The positions of the needle tips are marked in yellow. (TIF) [file ppat.1006118.s008.tif]

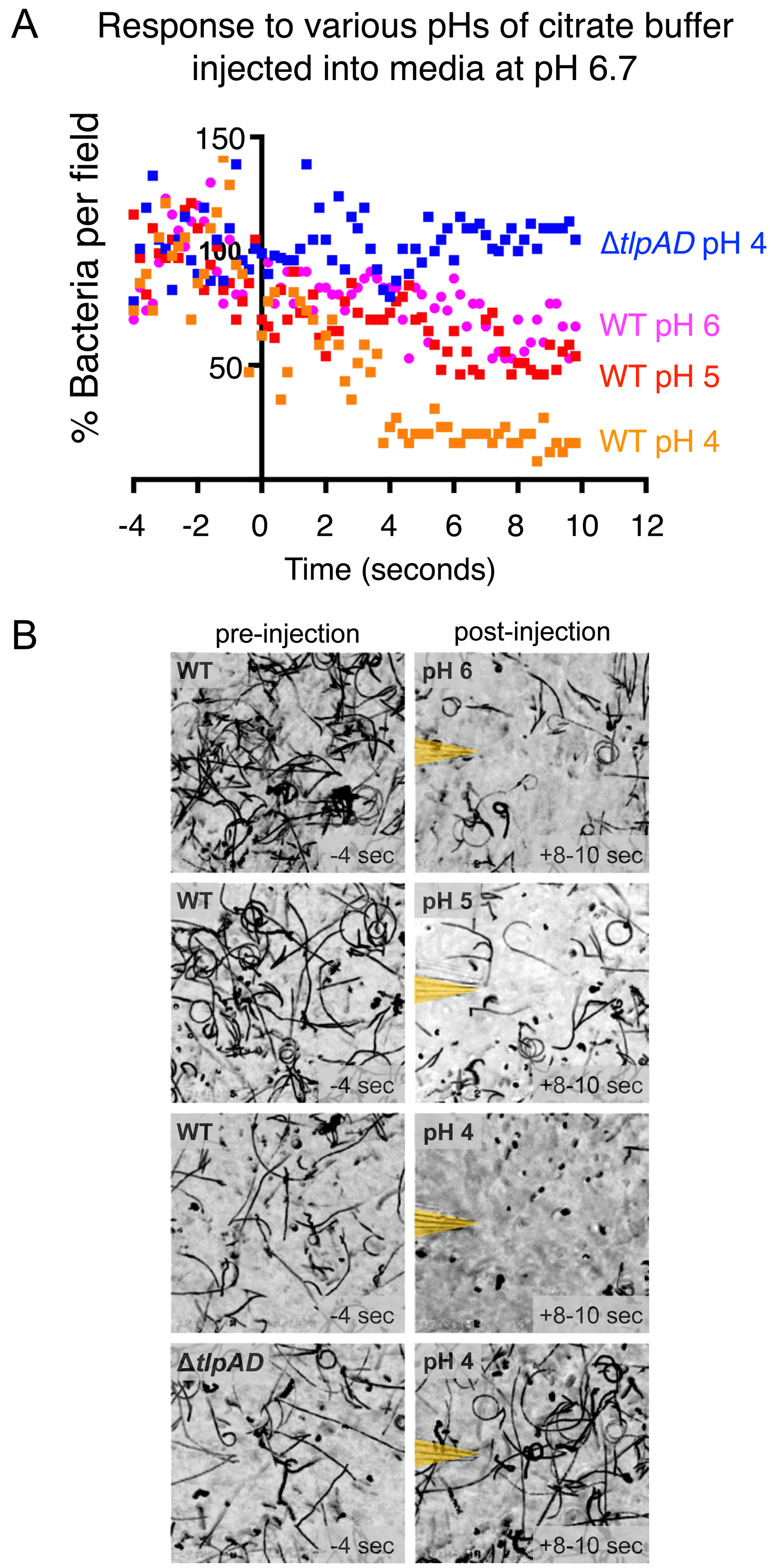

Supplement: S9 Fig — (A) Quantification of the responses of WT H. pylori to pH 4, 5, 6 buffered by citrate buffer. The response of ΔtlpAD to pH 4 is also shown. Each point represents the percent of swimming bacteria remaining in the field of view at each time point in the digitized video microscopy movie frames. Points for one representative movie are plotted per strain. Time zero is defined as the moment the needle is introduced and the gradient is initiated. (B) Still images of WT or ΔtlpAD motility traces lasting 2 seconds pre- and post- injection of citrate buffer at various pHs, from movies quantified in A. The positions of the needle tips are marked in yellow. (TIF) [file ppat.1006118.s009.tif]

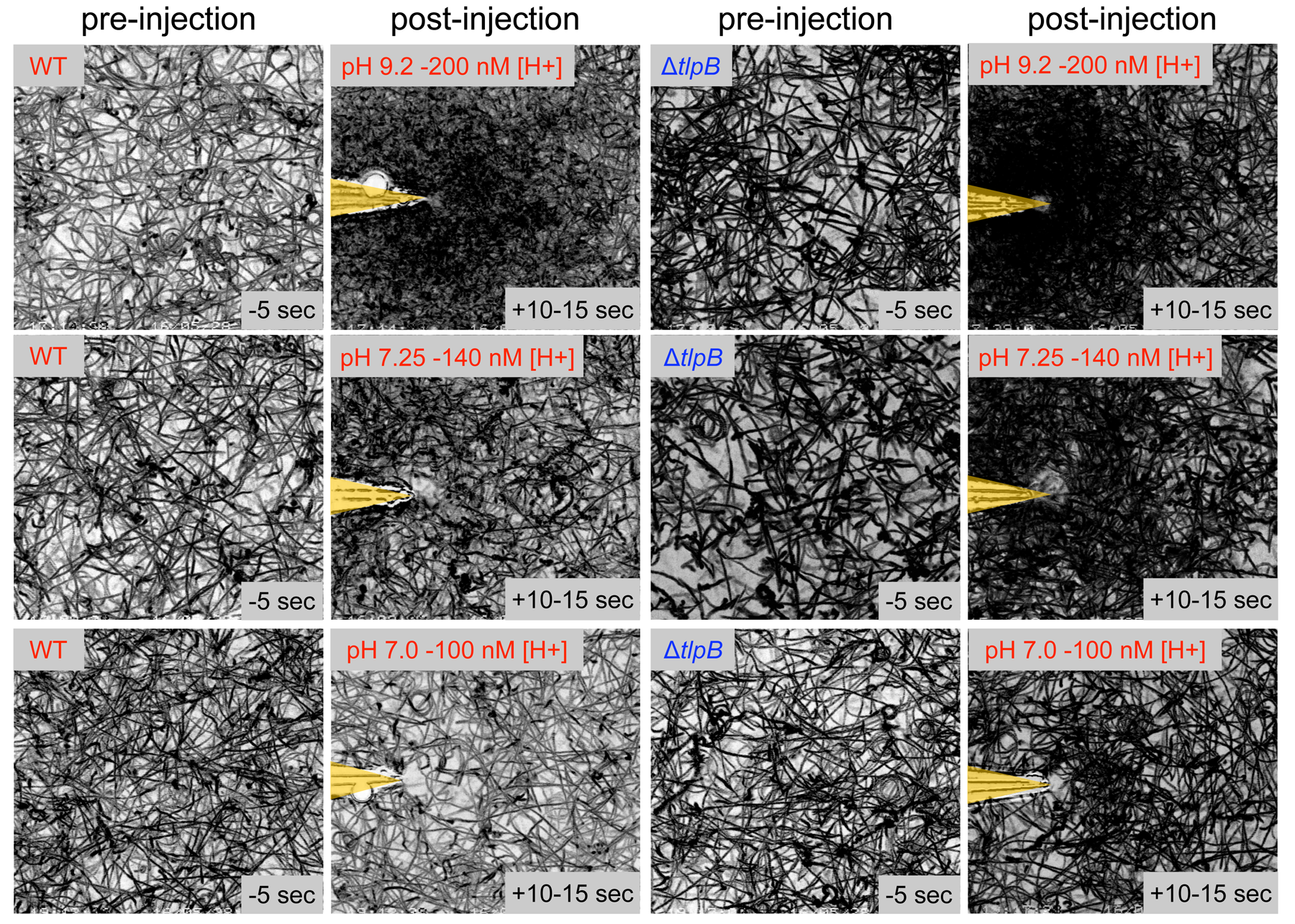

Supplement: S10 Fig — Still images of bacterial motility traces (lasting 5 seconds) of wild-type H. pylori vs. ΔtlpB before (panels in first and third columns, respectively) and after injection of phosphate buffer solutions with pH 9.2, 7.25, or 7.0 (panels in second and fourth columns, respectively). The pH of the culture medium was 6.7. The positions of the needle tips are marked in yellow. (TIF) [file ppat.1006118.s010.tif]

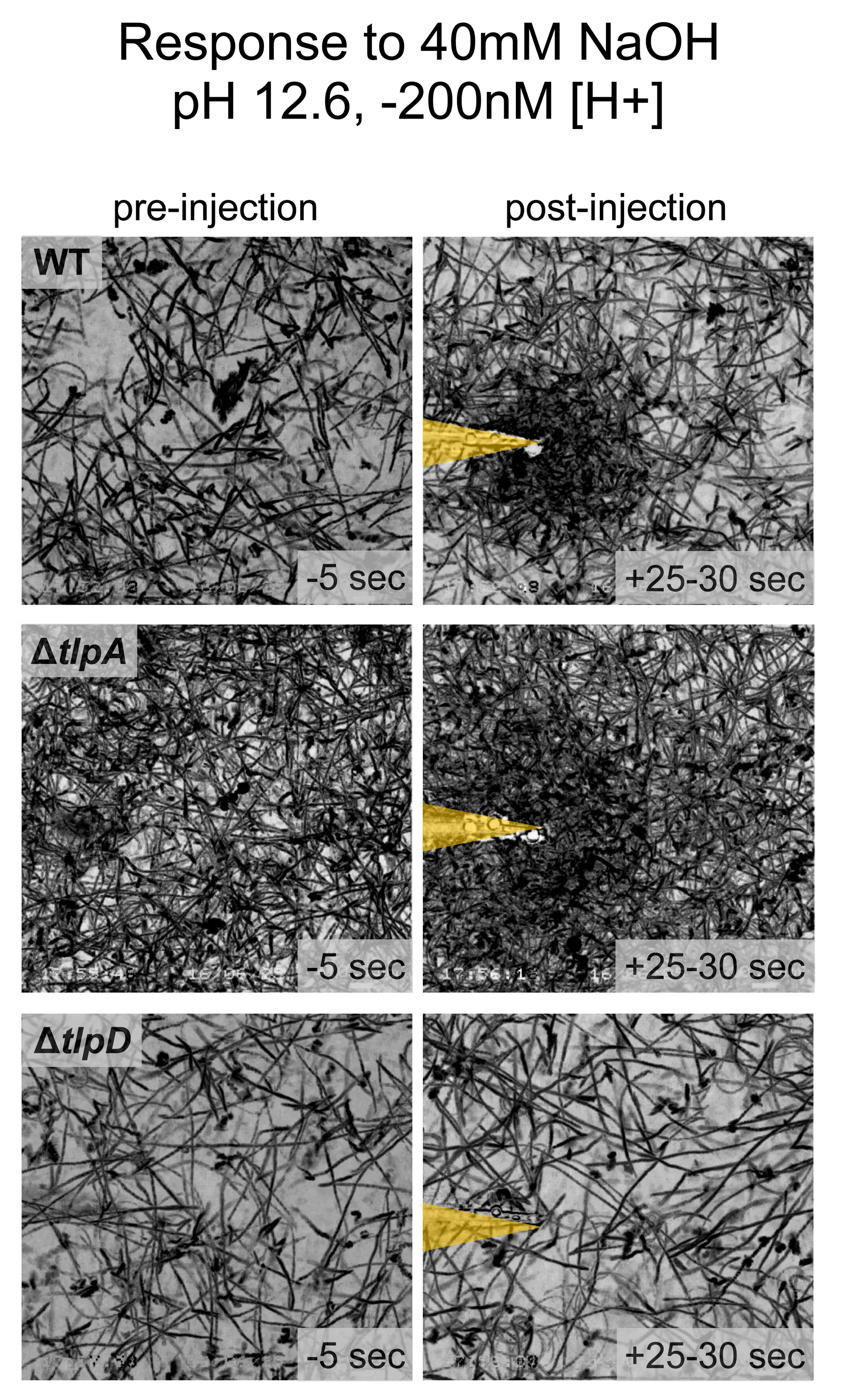

Supplement: S11 Fig — Still images of bacterial motility traces (lasting 5 seconds) of wild-type H. pylori vs. ΔtlpA vs. ΔtlpD before (panels in left column) and after injection of a 40mM sodium hydroxide solution with pH 12.6 (panels in right column). The pH of the culture medium was 6.7. The positions of the needle tips are marked in yellow. (TIF) [file ppat.1006118.s011.tif]

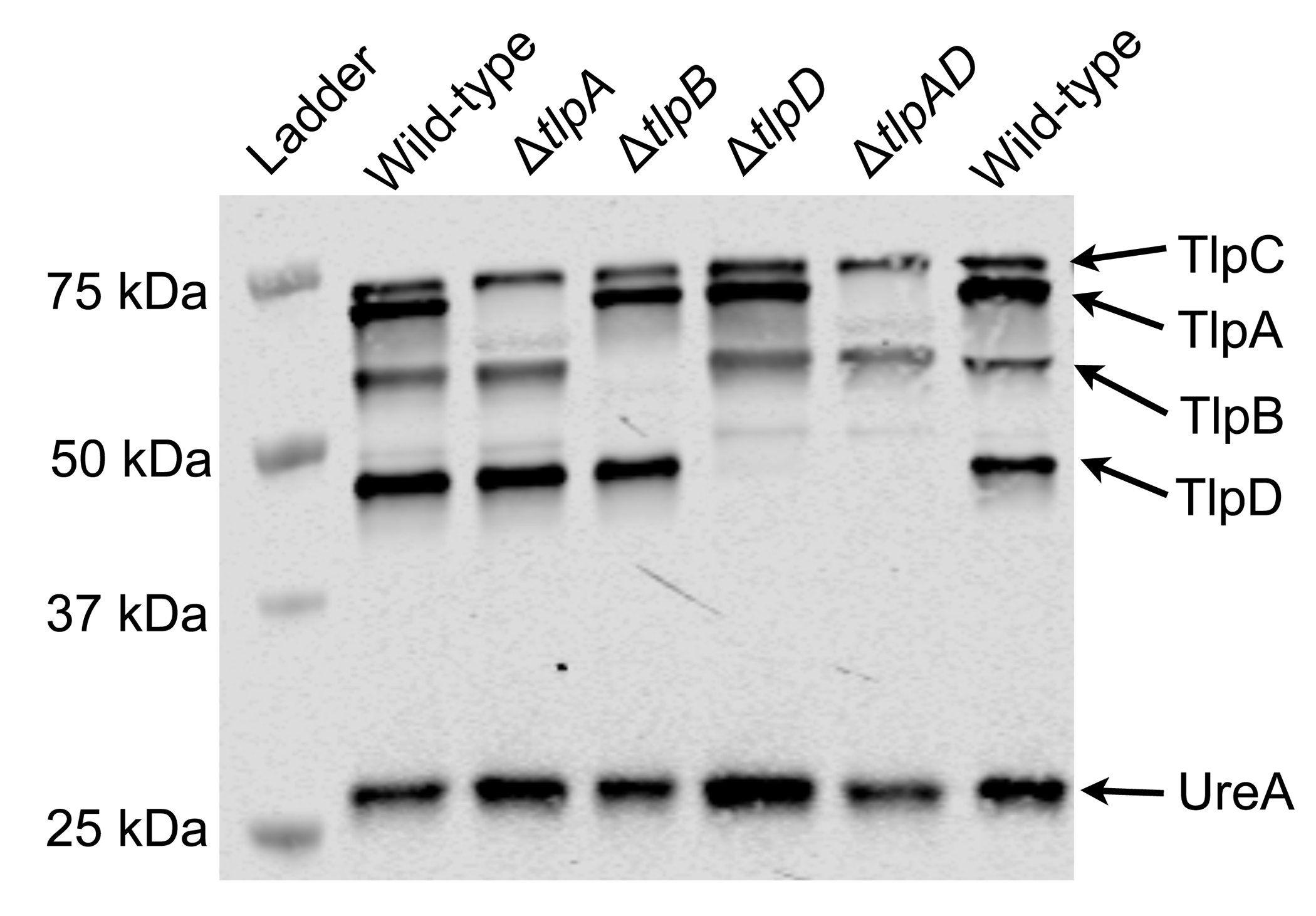

Supplement: S12 Fig — Immunoblot of H. pylori chemoreceptors from strain PMSS1. Whole cell SDS-SB lysates of ΔtlpA, ΔtlpB, ΔtlpC, ΔtlpD, ΔtlpAD and WT H. pylori were analyzed. An antibody that recognizes a conserved domain in all four chemoreceptors was used. UreA, the smaller subunit of urease, was used as a loading control. The band corresponding to each chemoreceptor and UreA are indicated. (TIF) [file ppat.1006118.s012.tif]

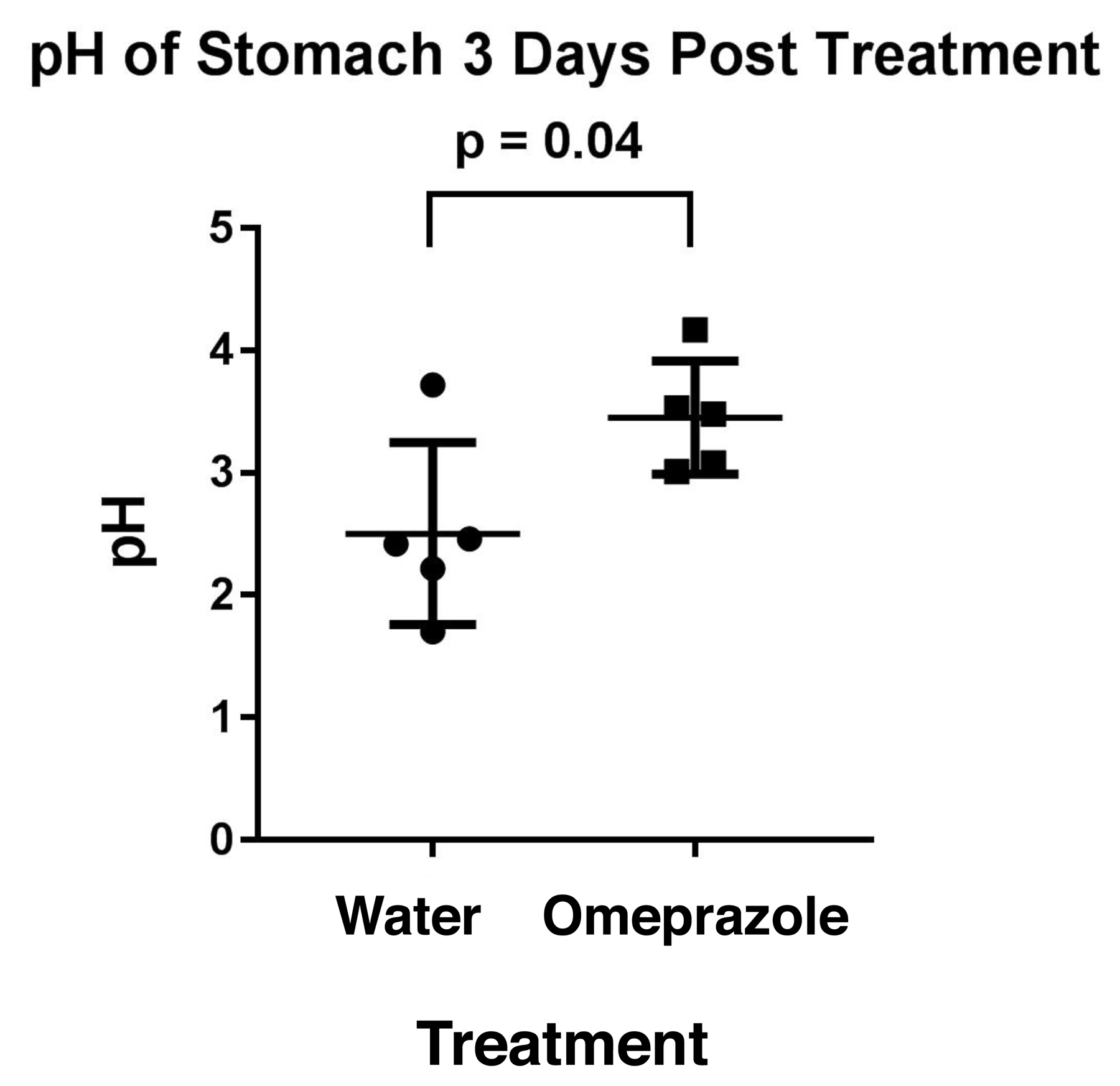

Supplement: S13 Fig — Omeprazole was administered in the drinking water as described in the Methods section. Food was not restricted. After 3 days of treatment, five mice were sequentially euthanized with carbon dioxide and the stomach immediately removed, opened through the lesser curvature and placed in 1 ml of distilled water. The stomach contents were mixed with the water and a pH probe inserted for measurements. The pH of stomachs harvested from 5 mice administered omeprazole in their drinking waters for 3 days versus 5 untreated mice are plotted. Significance was assessed by a Mann Whitney test. (TIF) [file ppat.1006118.s013.tif]

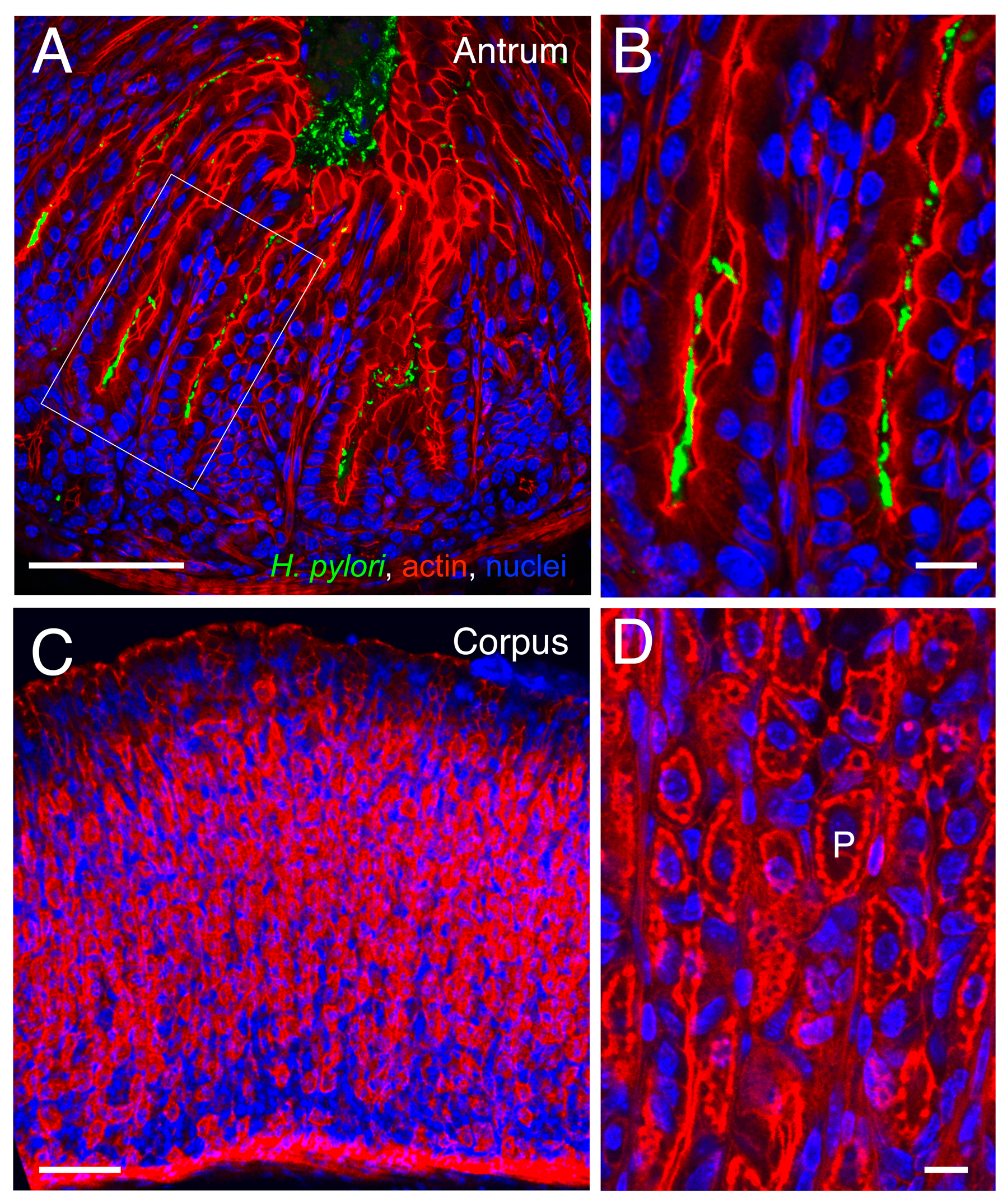

Supplement: S14 Fig — (A-B) 3D confocal immunofluorescence reconstruction of a region of the antrum of a mouse infected with wild-type H. pylori for two weeks. The boxed area is magnified in (B). (C- D) Corpus of the same mouse in low (C) and high (D) magnification. Parietal cells can be recognized by their large size and actin staining of the canalicular invaginations (P). H. pylori are absent from the corpus glands. H. pylori are stained with antibodies in green. The glands are visualized with actin staining with phalloidin in red and nuclei are stained with DAPI in blue. Scale bar is 100μm in A, C and 10μm in B, D. (TIF) [file ppat.1006118.s014.tif]
